# CFD-Assisted Process Optimization of an Integrated Photocatalytic Membrane System for Water Treatment

**DOI:** 10.3390/membranes13100827

**Published:** 2023-10-09

**Authors:** Vimbainashe Mercy Chakachaka, Charmaine Sesethu Tshangana, Bhekie Brilliance Mamba, Adolph Anga Muleja

**Affiliations:** Institute for Nanotechnology and Water Sustainability, College of Science, Engineering and Technology, University of South Africa, Florida Science Campus, Johannesburg 1709, South Africa

**Keywords:** reactor performance, simulated flow field, convective mass flow, mass transfer limitations, mixing speed, pharmaceutical photodegradation

## Abstract

An integrated photocatalytic membrane system (IPMS) was developed for potential use in the remediation of naproxen using real water samples from a drinking water treatment plant. Key parameters such as time, pH, water matrix, mixing speeds, flow rate, and light intensity undeniably affected photocatalytic and membrane separation processes. The system optimization was based on improving irradiation to generate a more reactive species and mass transfer to increase the reaction rate. Upon optimization, IPMS achieved 99% naproxen removal efficiency. Computational fluid dynamics (CFD) simulated the flow patterns and radiation distribution inside the photocatalytic membrane reactor to improve irradiation and mass transfer during operation. The simulated flow field revealed the presence of dead zones with different velocities in the photocatalytic membrane reactor; this limited the mass transfer of reactive species in the reactor, resulting in uneven distribution of reactive radicals. The dead zones were mitigated by increasing the mixing speed, and as a result, convective mass flow improved process performance. The governing parameters (flow patterns and radiation distribution) of the simulated and experimental data were in agreement. The absorption of irradiation by the active site of the membranes improved with light intensity; at higher light intensities, the light irradiated deeper into the membrane. As such, the CoFe_2_O_4_ nanoparticles incorporated inside the membrane pores became highly activated, thus enhancing degradation. The obtained space–time yield (STY) (1.23 × 10^11^ mol/cm^2^.s) and photocatalytic space–time yield (PSTY) (4.39 × 10^11^ mol/W.s) showed that the developed IPMS was efficient regarding energy intensiveness and throughput for treatment of pollutants in water.

## 1. Introduction

Photocatalytic degradation and membrane separation have emerged as the most promising technologies in removing recalcitrant pollutants such as pharmaceuticals [1,2,3,4]. However, these technologies might not achieve the desired efficiency when applied as a stand-alone, necessitating additional treatment processes [4,5]. Integrating membrane separation and photocatalytic degradation can couple the constituent technology’s advantages while removing their drawbacks [4]. The relevance of photocatalysis is attributed to its ability to mineralize pollutants unselectively or, at the very least, degrade pollutants into less harmful products using reactive radicals [6,7]. Despite extensive research in the field of photocatalysis, its practical applications still face limitations, primarily due to the challenge of designing efficient reactors. Two types of reactors are commonly used: slurry reactors with suspended catalyst nanoparticles and reactors with immobilized nanoparticles. Slurry reactors generally exhibit higher activity due to their larger illuminated surface area. However, they come with drawbacks, such as the need for additional steps to recover catalyst particles, the potential release of photocatalysts into the water, issues related to light scattering, and sludge generation [6,7,8,9]. In contrast, previous research efforts have focused on increasing the light efficiency for photocatalytic reactions by altering the properties of the catalyst material to absorb light at longer wavelengths or by examining various light sources [6,7]. Knowledge of another crucial aspect of photocatalysis, the irradiation distribution in the photocatalytic reactor, remains lacking [10,11]. While the literature is inundated with a wide range of spatial irradiation distributions, intensities, and wavelengths [8,9], a lack of understanding of the radiation field distribution in photocatalytic reactors can result in incorrect conclusions about photocatalytic processes. The immobilization of photocatalysts can offer solutions to address the challenges associated with slurry photocatalysis [12,13,14]. In immobilized systems, light distribution is typically more homogeneous because interface scattering due to roughness is often neglected, and bulk scattering is not relevant due to absorption [15,16]. Studies have shown that uniform irradiation distribution in immobilized reactors can enhance the reaction rate. Membranes play a crucial role in these systems, supporting photocatalysts with or without filtration systems [1,17]. Adding photocatalytic nanoparticles to membranes endows them with photocatalytic properties, solute rejection capabilities, and self-cleaning abilities [18,19]. However, these systems still face limitations, including low photocatalytic activity and mass transfer restrictions owing to the relatively low surface area compared to slurry systems [20]. Optimizing the mass transfer process and irradiation distribution during operation is essential to overcome these limitations. Despite the potential of photocatalytic membrane reactors, comprehensive research and optimization in mass transfer modeling are still lacking. Additionally, the reported mass transfer correlations in the literature exhibit significant discrepancies attributed to variations in local flow behavior. As far as we can tell, investigation of the role of irradiation distribution and light intensity on the performance of CoFe_2_O_4_-PES membranes-based photocatalytic batch reactor has not been previously recorded in the open literature. This exploration is crucial for optimizing the design and operation of photocatalytic reactors, as it allows the identification of key factors that influence the overall performance. Furthermore, the scope of this work considers the role of mass transfer and fluid dynamics on the performance of photocatalytic membrane systems without a flow-through mechanism.

It is paramount to understand the development of fluid flow along the reactor channel to interpret mass transfer data accurately and develop effective strategies for successful scale-up to pilot plant and industrial applications.

In line with improving the performance of immobilized photocatalytic systems, the current work seeks to address ways to enhance irradiation distribution and surface-to-volume ratio through mass transfer modeling while relating the transport phenomena and the photocatalytic reaction on the membrane surface. The main aim is to investigate the influence of hydrodynamics on the mass transfer rate by solving both the momentum and mass transport equations simultaneously. This approach seeks to improve our understanding of the underlying phenomena taking place inside the reactor and provide a computationally efficient method for evaluating local mass transfer. By combining these equations, we can gain valuable insights into the interaction between fluid flow and mass transfer, allowing us to assess mass transfer rates more effectively while minimizing the need for extensive computational resources. Additionally, light propagation from the source to the membrane, the optical properties, and distribution were also considered. Studies on the role of irradiation and light intensity of a CoFe_2_O_4_-PES membrane photocatalytic batch reactor and membrane-integrated system are not readily available in the open literature.

In this study, a drinking water system composed of an integrated photocatalytic and membrane separation process was developed and investigated for its potential application in the water treatment process. A photocatalytic membrane reactor was employed as a pre-treatment process connected to a membrane filtration unit as a final treatment process. In the photocatalytic unit, the membrane acts as a pseudo-fixed catalytic layer, where the membrane serves as a support for photocatalytic nanoparticles without sieving. Each unit was optimized prior to the investigation of the performance of the IMPS. In the photocatalytic membrane reactor unit, focus was placed on (i) the fluid hydrodynamics, (ii) mass transfer, (iii) light intensity, and (iv) its distribution to find ways to mitigate mass transfer limitations and inaccessibility of the irradiation to the photocatalysts using CFD. This work paves the way and sheds light on the CFD simulation and investigation of the effect of irradiation distribution and light intensity for the performance of a CoFe_2_O_4_-PES membranes-based photocatalytic batch reactor on naproxen-contaminated water treatment.

## 2. Numerical Modeling

### 2.1. Mathematical Model and Simulation

Computational modeling was conducted using the SimScale CFD package. SimScale is a cloud-based simulation software known for its advantages in CFD studies. Its user-friendly web interface provides easy accessibility and collaboration, eliminating complex installations. With powerful remote computing resources, SimScale reduces the need for expensive hardware and enables the execution of complex simulations. The CFD was applied to understand the flow behavior of the reaction mixture during mixing and to relate obtained mass transfer patterns to IPMS’ performance.

#### 2.1.1. Hydrodynamic Model

Although hydrodynamic behavior simulation of the IPMS can be performed using different techniques, the use of turbulence models coupled to Reynolds-averaged Navier–Stokes equations (RANS) is an effective and practical tool for this class of problems. Assuming negligible variations in viscosity and density, the RANS model employs momentum Equation (1) and mass (Equation (2)) conservation equations.

(1) $$\frac{\partial }{\partial t}\left(\rho U_{j}\right)+\frac{\partial }{\partial x_{i}}\left(\rho U_{i}U_{j}\right)=-\frac{\partial P}{\partial x_{j}}+\frac{\partial }{\partial x_{i}}\mu \left(\frac{\partial U_{i}}{\partial x_{j}}+\frac{\partial U_{j}}{\partial x_{i}}\right)+\rho g_{j}+F_{j}$$

(2) $$\frac{\partial \rho }{\partial t}+\frac{\partial U_{i}}{\partial x_{i}}=0$$

where t is the time, $x_{i}$ denotes the *i* spatial coordinate, $x_{j}$ is the *j* spatial coordinate, $U_{i}$ and $U_{j}$ are the *i* and *j* components of the fluid velocity $U_{\;},$ ρ is the gravitational acceleration, P and F are pressure, and the external force per unit volume.

Under turbulent flow, the concentration and velocity fluctuation terms can be grouped into a so-called turbulent diffusion term of an averaged diffusion–convection equation analogous to the RANS equation. Mass transport was modeled using the k-omega (ω) turbulence. A k-ω turbulence model involves solving the transport equation for a turbulent diffusivity coefficient (k-omega) to simulate the distribution and transport of species in a fluid flow.

K-omega Transport Equations: The k-omega turbulence model introduces two additional transport equations to solve for the turbulent kinetic energy (*k*) and the specific turbulent dissipation rate (omega).

Transport equation for *k*:

(3) $$\frac{\partial }{\partial t}\left(\rho k\right)+\frac{\partial }{\partial x_{j}}\left(\rho U_{j}k\right)=\mu_{t}\left(\frac{\partial U_{i}}{\partial x_{j}}+\frac{\partial U_{j}}{\partial x_{i}}\right)\frac{\partial U_{i}}{\partial x_{j}}-\beta^{*}\rho \omega k+\frac{\partial }{\partial x_{j}}\left(\left(\mu +\frac{\mu_{t}}{\sigma_{k}}\right)\frac{\partial k}{\partial x_{j}}\right)$$

Transport equation for omega:

(4) $$\frac{\partial }{\partial t}\left(\rho \omega \right)+\frac{\partial }{\partial x_{j}}\left(\rho U_{j}\omega \right)=C_{1}\mu_{t}\frac{\omega }{k}\left(\frac{\partial U_{i}}{\partial x_{j}}+\frac{\partial U_{j}}{\partial x_{i}}\right)\frac{\partial U_{i}}{\partial x_{j}}-\beta \rho \omega^{2}+\frac{\partial }{\partial x_{j}}\left(\left(\mu +\frac{\mu_{t}}{\sigma_{\omega }}\right)\frac{\partial \omega }{\partial x_{j}}\right)$$

where k denotes the turbulent kinetic energy, ω is the specific dissipation rate of k, ∂k, ∂ω, $C_{1}$, β, and $\beta^{*}$. A turbulent diffusivity coefficient (*D_t_*), a mass transfer property, can be related to the turbulent kinetic energy (*k*) using Equation (5).

(5) $$D_{t}=\frac{C_{D}}{SC_{T}}.\frac{k^{2}}{g}$$

where $C_{D}$ is a constant, $SC_{T}$ is the turbulent Schmidt number, and g is the energy dissipation rate. The advection–diffusion equation for the concentration (*C*) of that species can be solved using Equation (6) to model the transport of a species.

(6) $$\frac{\partial \rho C}{\partial t}\nabla .\left(\rho C_{\;}\right)=\nabla .\left[\left(D+D_{t}\right)\left(\nabla C\right)\right]$$

where *D* is the molecular diffusivity of the species.

#### 2.1.2. Radiation Transport Model

Regarding radiation transfer, CFD-aided radiation simulations were performed based on the Monte Carlo Model. The Monte Carlo Model was used to solve the radiation transport equation (RTE) (Equation (7)). RTE offers an opportunity to compute the local volumetric rate of photon absorption and local volumetric rate of energy [14]. The local volumetric rate of energy absorption (LVREA) depends on the photon distribution in the reaction medium.

(7) $$\frac{dI_{\lambda }\left(S,\;\Omega \right)}{ds}=-K_{\lambda }I_{\lambda }\left(s,\;\Omega \right)-\sigma_{\lambda }I_{\lambda }\left(s,\;\Omega \right)+\frac{1}{4\pi }\sigma_{\lambda }\int_{0}^{4\pi }p\left(\;\Omega^{\prime }\to \Omega \right)I_{\lambda }\left(s,\;\Omega^{\prime }\right)d\;\Omega^{\prime })$$

where $I_{\lambda }$ is the specific light intensity of a ray at wavelength *λ* traveling a distance *s* in direction Ω, $p\left(\;\Omega^{\prime }\to \Omega \right)$ is the scattering phase function, and $\sigma_{\lambda }$ and $K_{\lambda }\;$ are the scattering and absorption coefficients. The absorption probability of irradiation was evaluated by employing inequality, as shown in Equation (8).

(8) $$\frac{K_{\lambda }}{K_{\lambda }+\sigma_{\lambda }}>R_{\;}$$

where $R_{1}$ represents a random number uniformly distributed between 0 and 1. Media such as water and air were considered transparent and had no interactions with radiation, and the emission of radiation was assumed negligible at low-temperature operation. The irradiation uniformity index was also employed to evaluate the uniformity of light intensity across the surface of the photocatalytic membrane. The uniformity of the light distribution was assessed using the area-weighted uniformity index, γ, provided by Equation (9).

(9) $$\gamma =1-\frac{\sum_{i=1}^{n}A_{i}/\emptyset_{i}-\emptyset_{av}/}{2\emptyset_{av}\sum_{i=1}^{n}A_{i}}$$

where $\emptyset_{av}$ is the average light intensity and $\emptyset_{i}$ is the local light intensity for pixel *i* with the pixel area $A_{i}$.

#### 2.1.3. Simulations

**Figure 1:**
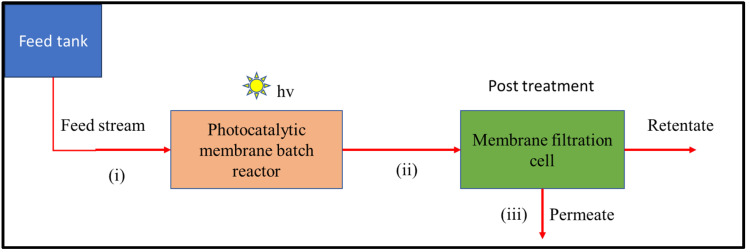
The process flow sheet of the IPMS was employed to remove naproxen.

The CFD model was developed to simulate the IPMS using the commercial software SimScale (https://www.simscale.com). The purpose of employing the CFD approach was to investigate the flow behavior of the reaction mixture during the mixing process and to establish a connection between the observed mass transfer patterns and the performance of the IPMS. The IPMS was a two-stage process composed of two units: a photocatalytic membrane batch reactor and a membrane filtration system. The two units were connected in series to produce a single continuous flow mode (Figure 1).

The photocatalytic membrane batch reactor was employed as a pre-treatment step for effectively degrading recalcitrant pollutants and potential foulants before the water undergoes membrane filtration in the final stage. The membrane filtration unit served as the final treatment stage (before disinfection in a real water treatment plant context), facilitating the retention of any un-oxidized organic pollutants from the pre-treatment stage. By employing membrane filtration as the final step, the integrated system ensured that any remaining pollutants that were not completely degraded or mineralized during the photocatalytic process were effectively removed and prevented from spreading further.

**Figure 2:**
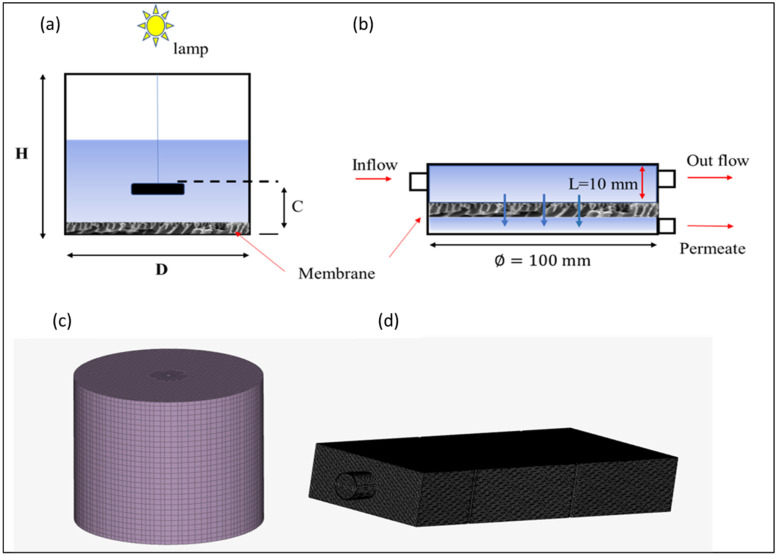
Virtual representations of (**a**) the photocatalytic reactor dimensions and (**b**) the membrane filtration dimensions, (**c**) the mesh for the photocatalytic reactor, and (**d**) the mesh for the membrane filtration unit.

Both units housed flat-sheet CoFe_2_O_4_-PES nanocomposite membranes with 0.01 mm thickness and surface areas of 5 × 10^3^ mm^2^. In the photocatalytic membrane batch reactor, light was irradiated to facilitate photocatalytic degradation. While in the membrane filtration unit, no light was introduced, and the pollutant removal mechanism was strictly filtration driven by pressure. The role of the membrane in the batch reactor was solely to support the photocatalysts. In the batch reactor, the membrane was configured to facilitate the fluid interaction with the photocatalyst by diffusion and convective mass transfer. The IPMS units were defined to meet the dimensions and characteristics of the experimental system (Figure 2) using SimScale’s built-in browser-based CAD tool.

The geometry of the photocatalytic membrane batch reactor was cylindrical with a diameter (D of 100 mm) and a height (H) of 200 mm, as shown in Figure 2a. In the photocatalytic membrane batch reactor, the mixer was located at an axial position (C) of 10 mm from the bottom and had a diameter of D/3. The light source was situated at the top of the reactor. The membrane was configured as shown in Figure 2a.

For the membrane separation unit, the water layer on top of the membrane had a thickness of 10 mm (Figure 2b). The inflow tubing was positioned 5 mm from the side wall and had a diameter of 10 mm. The thickness of the water layer behind the membrane was 0.3 mm. The outflow tube was located at the bottom and positioned in the center of the membrane separation unit. The outflow tube was similar to the inflow tube, and the filtration unit operated under crossflow mode.

Based on the pollutant removal mechanism of the IPMS, the study aimed to model the hydrodynamic characteristics and radiation distribution of the photocatalytic membrane batch reactor and the flow patterns in the filtration unit.

SimScale was employed to model the fluid dynamics inside the membrane batch reactor. The system was simulated under isothermal and steady-state conditions. The polluted water stream was considered a Newtonian incompressible fluid. The mathematical modeling of the system was then expressed through the solution of mass, momentum, and species conservation equations (Equations (1)–(6)). A virtual model of the whole geometry (batch reactor) was built, and the domain was discretized into sufficient volume elements. A grid independence test was performed by simulating the reactor with different mesh sizes. The model reactor geometry and mesh are represented in Figure 2. To discretize the computational domain of the reactor, it was divided into two distinct sections. The inner region, which housed the rotating mixer, was discretized using tetrahedral grids, while the stationary zone was discretized using hexahedral grids. To solve the CFD model for the fluid hydrodynamic in the batch reactor, the boundary of the rotating domain was set within the range of $\frac{C}{2}\leq z\geq 3\frac{C}{2}$. C denotes the length between the mixer and the membrane mounted at the surface of the reactor; see Figure 2a. The reactor’s three-dimensional coordinate system originates at the bottom center. The liquid’s top surface is symmetric, and the solid walls impose a no-slip boundary condition. A user-defined function was employed to enhance simulation efficiency. A user-defined interface was employed to reduce the computational time. The mass transfer model during mixing was solved in the time domain with the second-order implicit time difference format after the steady-state flow field was obtained and $\rho_{1}=0.0,\;\rho_{1}U_{1}=\rho_{2}U_{2}=0.0$ were set to resolve the model using the user-defined interface. Kinetics of naproxen were not considered in simulation studies.

Pertaining radiation, the objective of the simulation was to analyze the distribution and intensity of light within the photocatalytic membrane batch reactor to ensure optimal activation of the photocatalyst for the desired reaction. The modeling approach focused on obtaining the incident radiation flux over the photocatalytic surfaces using a CFD model by the resolution of the RTE (Equation (7)). The light source, a Xenon lamp with a bulb rating of 300 W and an apparent power of 500 VA, was placed on top of the reactor in the simulation setup (Figure 2a). The emission system was comprised of (i) the lamp, (ii) the vessel geometry, and (iii) the spatial distribution of the lamp in the vessel. Therefore, two boundary conditions were established. External boundary conditions were set using the nominal output power provided by the lamp manufacturer; for the internal boundary, which is comprised of the surface of the photocatalytic CoFe_2_O_4_-PES membrane, the absorption coefficient and reflectivity were defined. The absorption coefficient, obtained experimentally from UV-Vis absorbance data, was determined to be 10^−4^ cm^−1^. The lamp was defined as a purely diffuse surface with an external irradiance given by the manufacturer. All the other surfaces in the domain were considered non-reflective. The space between the lamp and the reactor window, basically air, was treated as a diactinic medium (no absorption, scattering, or emission). An unstructured mesh was generated using the meshing tool, discretizing the batch reactor and the illumination system into numerous finite-volume elements. Additionally, a finer mesh was employed near the lamp unit and over the catalytic surfaces to capture the details of the radiation source and the photocatalytic regions. The non-sequential ray-tracing algorithm was based on the Monte Carlo method and the principles of ray optics.

For the membrane filtration unit, the role of fluid dynamics on filtration performance was investigated using Reynolds-averaged Navier–Stokes equations. Prism-based boundary layers were used to mesh the membrane–water interfaces, top and bottom walls, and other nearby surfaces. A very fine grid (*y* + 1) was made to ensure the thin boundary layer was set correctly, with the first grid node close to the membrane at 1 × 10^4^ mm [12].

#### 2.1.4. Operating Conditions

In the photocatalytic membrane batch reactor, physical mixing was achieved with a mixer operating at 150, 250, 300, 450, 500, and 550 rpm. The corresponding Reynolds numbers were in the range of 6240 to 22,876. The diffusion coefficient of naproxen was set to 5.7 × 10^−10^ m^2^ s ^−1^. The physical properties of water used were a density of 1000 kg/m^3^, thermal conductivity of 0.6 W/(m·K) at 25 °C, and kinematic viscosity of 1 × 10^−6^ m^2^/s. For air, density was 1.225 kg/m^3^, thermal conductivity was 0.0257 W/(m·K), and kinematic viscosity was 1.5 × 10^−5^ m^2^/s. The free surface at the top of the liquid in the reactor was defined to be symmetrical, and all solid walls enforced the no-slip boundary conditions. Naproxen was initially added as part of the feed solution (contaminated water) before resolving the fluid dynamics. Further, naproxen had no significant effect on the fluid dynamic of the system because it imposed an insignificant effect on the density of water.

The radiation model for the batch reactor required key simulation parameters, including the reactor’s shape and dimensions, the optical properties of the materials used, and appropriate boundary conditions for resolving the radiative transfer equation (RTE). The chosen light source was the HAL-320 Compact Xenon lamp with a bulb rating of 300 W and an apparent power of 500 VA. A surface emission model was employed, treating the lamp as a purely diffuse surface with external irradiance specified by the manufacturer. All other surfaces within the domain were considered non-reflective. The space between the lamp and the reactor window, which consisted of air, was treated as a diactinic medium with no absorption, scattering, or emission. The reactant fluid was also treated as diactinic. The convergence criteria of the steady-state simulations were set as 10^−3^ for the momentum and 10^−5^ for continuity and water species. Details of ray tracing and optimization are provided in Section S5 of the Supplementary Material (Figure S3).

The fluid separation was achieved through a crossflow filtration mechanism in the membrane filtration unit. The contaminant removal mechanism was based on molecular sieving without photocatalytic reaction. Hence, light distribution simulations were performed only for the batch reactor system. The filtration unit operated under crossflow mode. The porosity and permeability used were obtained experimentally from the fabricated membranes. The walls of the filtration unit were treated as no-slip boundaries for the flow and no flux for the transport. The inlet velocity was calculated from the feed flow of 10 L·h^−1^. The feed flow was selected from the experimental work. At the reactor outlet, the pressure was set at zero. The applied solver is a built-in COMSOL solver: a segregated solver for velocity, pressure, and concentration using an algebraic multigrid solver.

## 3. Experimental Procedures

### 3.1. Fabrication and Characterization of Membranes

Details of membrane fabrication and characterization are provided in Section S2 of the Supplementary Material.

### 3.2. Experimental Setup and Performance Tests

The integrated photocatalytic membrane system consisted of three main process streams connecting the IPMS into a single continuous flow mode: (i) the feed stream, (ii) the semi-batch reactor output stream (which was used as the feeding stream to the membrane filtration unit), and (iii) the permeate stream. Water samples collected from a South African water treatment plant were used during photodegradation and rejection investigations (real water parameters are given in the Supplementary Material in Table S1). Naproxen (5 mg/L) was used as a model pharmaceutical pollutant to evaluate the performance studies of IPMS. In the semi-batch reactor unit, irradiation was provided by a HAL-320 Compact Xenon light source with a bulb rating of 300 W and an apparent power of 500 VA.

This study equipped the photocatalytic membrane reactor system with a temperature regulator. This regulator played a critical role in maintaining temperature, ensuring that no significant variations in temperature were observed during the experimental process. During photodegradation, 5 mL aliquots were drawn from the reactor at pre-determined time intervals (0, 30, 60, 90, 120, and 150 min) using a syringe for UV-Vis spectrometer analysis. Loss of intensity and shift in the peak position at 230 nm was considered degradation.

Equation (10) was used to calculate the percentage of naproxen degraded.

(10) $$\%\;of\;naproxen\;degraded=\frac{C_{i}-C_{t}}{C_{i}}\times 100$$

where $C_{i}$ and $C_{t}$ are the initial naproxen concentration and naproxen concentration at time *t*.

For the filtration cell, a single-cell crossflow filtration setup was used to investigate the rejection properties of the IPMS, and the pressure was generated using a pump. Both the photocatalytic membrane batch reactor and membrane filtration unit employed CoFe_2_O_4_-PES membranes. Before performance studies were conducted, the membranes were compacted at 6 bar until a steady flux was obtained. After membrane compaction, water fluxes of the membranes were measured. Equation (11) was used to determine the permeate flux of each membrane.

(11) $$J_{w}=\frac{V}{At}\;$$

where $J_{w}\;$ is the pure water flux (L/m^2^h), V is the volume of water (L), *A* is the surface area of the membrane (m^2^), and t is the collection time (h). Rejection of naproxen was investigated at 4 bar, and the concentration of naproxen was quantified using the TOC instrument. Rejection was calculated using Equation (12).

(12) $$R\%=\left(1-\frac{C_{p}}{C_{f}}\right)\times 100$$

## 4. Results and Discussion

### 4.1. Grid Independence Test for the Batch Reactor

The grid independence was assessed using the predicted power number [21]. Two calculations were performed to determine the power consumption (*P*) in this evaluation. Firstly, the power number ($P_{T})$ was computed using Equation (13), where torque was used to estimate the power number.

(13) $$P_{T}=2\pi MN$$

where *M* denotes the toque. Secondly, the power *P_ℇ_* was calculated by integrating the turbulent dissipation rate *ℇ* over the entire vessel volume, indicating the sensitivity of turbulent quantities to mesh density (Equation (14)).

(14) $$P_{ℇ}\;=\rho ∭ℇ\;\mathrm{dV}$$

After determining *P_ℇ_*, the power number *N_P_* was determined using Equation (15).

(15) $$N_{p}=\frac{P}{\rho N^{3}D^{5}}$$

**Figure 3:**
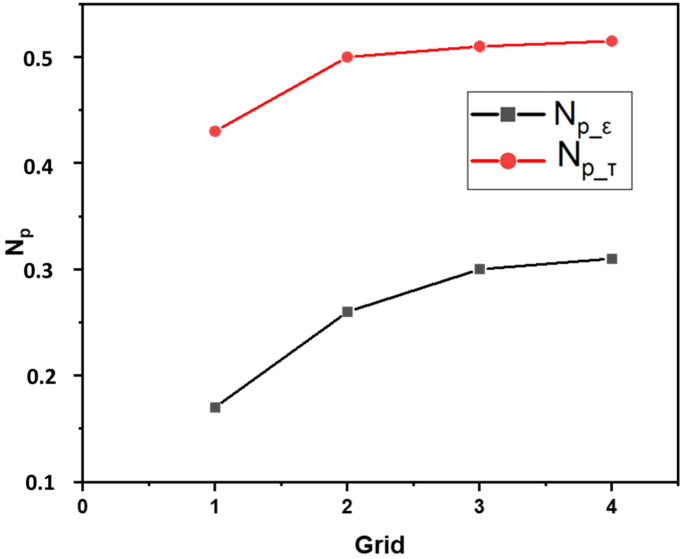
The effect of grid number on the predicted power number at 550 rpm.

For this study, different grid configurations were tested, where grid one consisted of approximately 0.4 million control cells, grid two of 0.85 million, grid three of 1.20 million, and grid four of 1.6 million control cells. The influence of the grid number on the predicted power number was investigated at the highest impeller speed of 550 rpm, and the results are presented in Figure 3. From a close analysis of Figure 3, it can be observed that the grid independence of $N_{p\_\tau }$, which is estimated using torque, is largely achieved with grid two. However, the calculated $N_{p\_\varepsilon }$ based on dissipation exhibits a slight increase from grid two to grid three. It is worth noting that $N_{p\_\varepsilon }$ consistently remained smaller than $N_{p\_\tau }$ for all four grid configurations, which is consistent with the findings reported by Coroneo et al. [21].

**Figure 4:**
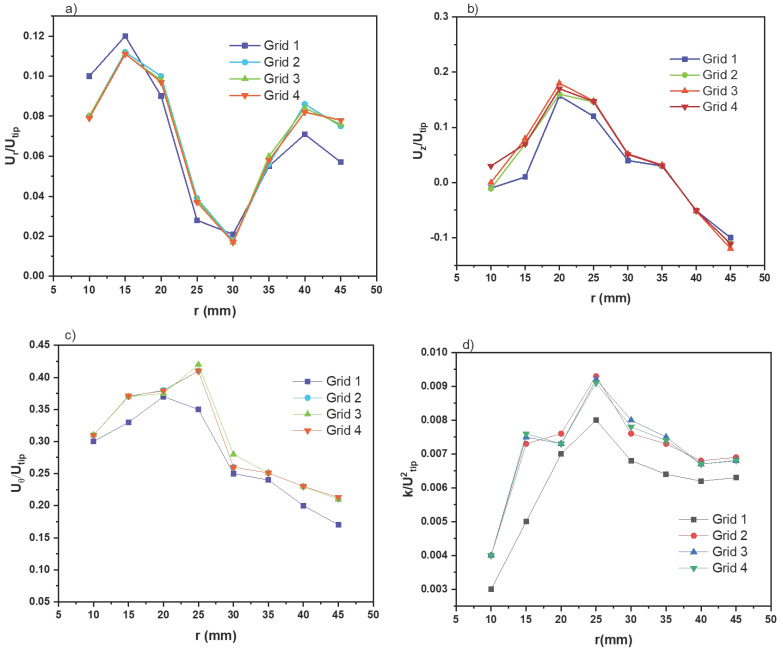
Radial profiles of the azimuthally averaged velocity components (**a**) radial velocity, (**b**) axial velocity, (**c**) tangential velocity, and (**d**) turbulent kinetic energy.

Figure 4 presents the radial profiles of azimuthally averaged dimensionless velocity components and turbulent kinetic energy. Despite the inability to eliminate the discrepancy between the two *N_P_* values with the increase in grid number, it was observed that the results obtained using grid three showed that grid independence could accurately capture the hydrodynamic characteristics of the photocatalytic membrane reactor. As a result, grid three, consisting of 1.20 million control cells, was selected and utilized in this study.

### 4.2. Simulation of Physical Mixing Inside the Batch Reactor

**Figure 5:**
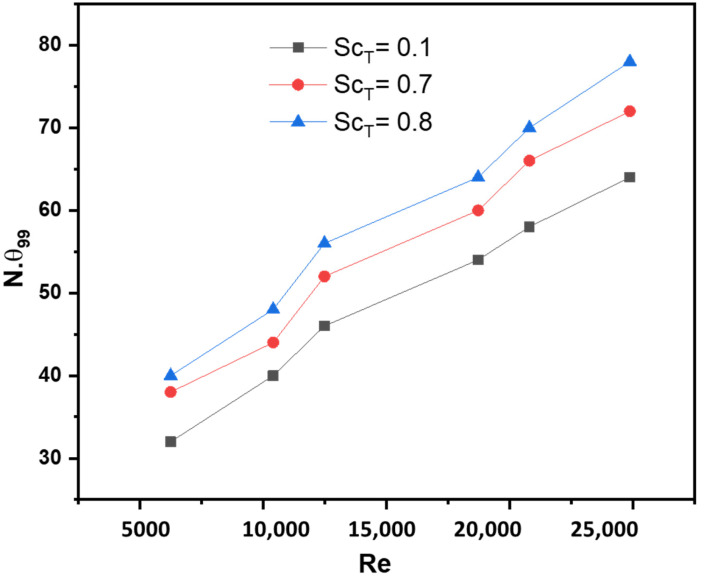
Role of mixing speeds on mixing time at different turbulent Schmidt numbers.

The evaluation of the macro-mixing process in the reactor relies heavily on the non-reactive mixing time. This parameter is determined by analyzing the temporal variation of the area-averaged tracer concentration on half of the cross-section (*y* = 0). To define the non-reaction mixing time (denoted as 99), Hu et al. [22] established the criterion of maintaining the mean concentration within the range of 99% to 101% of the final uniform value. Figure 5 displays the relationship between the predicted dimensionless mixing time (N_99_) and the Reynolds number.

The turbulent Schmidt number (Sc_T_) characterizes the difference between the momentum and passive scalars’ transport rates. Its value varies across different applications, typically within the range of 0.1 to 1.0, as chosen by researchers [23,24,25,26].

In this study, the analysis focused on the commonly employed values of 0.1, 0.7, and 0.8 for the turbulent Schmidt number. The relationship depicted in Figure 5 reveals that the physical mixing time increases when Sc_T_ is elevated. Moreover, the dimensionless mixing time (N_99_) tends to approach a stable value as the Reynolds number increases. At 550 rpm, the macro mixing time was calculated using Equation (16) as reported by Joshi et al. [27]:

(16) $$\theta =5.0\left(\frac{2H}{D}+\frac{T}{D}\right)\left(\frac{H-C}{D}\right)/\left(\frac{N}{60}\right)$$

The results showed that it takes 3.5 s to achieve a homogenous mixture in the reactor at 550 rpm.

### 4.3. Velocity Profiles Inside the Batch Reactor

**Figure 6:**
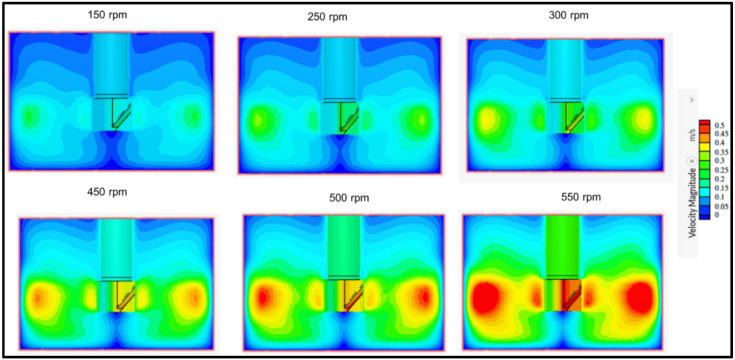
Simulated flow field at different mixing rates in the batch reactor used for naproxen removal at C = H/3.

The flow field in the batch reactor was investigated, and Figure 6 depicts the variation of the velocity magnitude at different rotating speeds from 150 to 550 rpm at (C = H/3). The spatial distribution of velocity showed that the velocity magnitude increased with the mixing speed. At 150 rpm and 250 rpm, the highest velocity zones are displayed by green contours. From the scale bar (Figure 6), a maximum of 0.2 m/s was attained via a small fraction of the reactive mixture close to the mixer, and the magnitude decreased towards the peripherals of the reactor. A similar pattern was observed previously in the literature [28,29]. At low mixing speeds, dead zones in the reactor were demarcated by dark-blue contours corresponding to the 0 and 0.05 m/s in the scale bar of Figure 6, which are distinguished by extremely low velocities. However, it was observed that as the speed increased from 150 to 300 rpm, a larger fraction of the reactive mixture gained up to 0.3 m/s velocity. The increase in velocity was anticipated to improve reaction efficacy by increasing the mass transfer of the reactive species.

On the other hand, when the mixing speed was above 450 rpm up to 550 rpm, the velocity increased from 0.45 m/s to 0.5 m/s, as marked by the appearance of red contours indicating strong interactions between water and reactive species. The dead zones decreased significantly with an increase in the velocity magnitude from 150 rpm to 550 rpm. Although the dead zones were reduced, high velocities may introduce vortices, chaotic eddies, and mixing instabilities, which can result in the decline of degradation performances. The latter was confirmed with experimental data reported in Section 4.6.5.

To further understand this flow field distribution in terms of mass transfer, the Sherwood number was commutated using Equation (17).

(17) $$Sh=Re^{0.5}.Sc_{T}{}^{0.33}$$

The Sherwood number (Sh) represents the dimensionless mass transfer coefficient, and a higher Sh number implies a higher efficiency or effectiveness of the mass transfer. The Sh values for mixing speeds 150, 250, 300, 450, 500, and 550 rpm corresponding to Reynolds numbers 6240, 10,400, 12,478, 18,717, 20,796, and 24,870 were 70.22, 90.65, 99.30, 121.61, 128.19, and 140.19, respectively. The increase in the Sh number can be attributed to factors such as increased turbulence and improved mixing. These factors facilitate a more efficient mass transfer between phases or across a boundary. The obtained trend implies that more mass is being transferred within a given timeframe, leading to better mixing through convection and diffusion, enhanced reaction rates, or improved overall performance of the system involving mass transfer processes.

### 4.4. Role of the Boundary Layer on Mass Transfer of Naproxen in the Batch and Membrane Separation Processes

**Figure 7:**
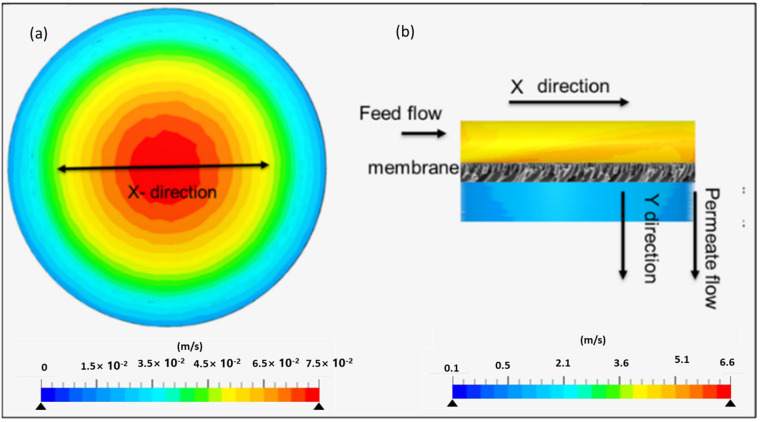
Velocity profile of the water matrix along the x plane in a (**a**) batch reactor and (**b**) membrane separation unit showing the fluid channel flowing through the membrane.

The role of a boundary layer on mass transfer and naproxen removal efficiency was analyzed using a simulated flow field. The obtained velocity profile described the hydrodynamic characteristics of water inside the process units (Figure 7), which is crucial for process optimization. In the batch reactor unit, the motion was achieved via a rotating stirrer with no defined direction, as shown in Figure 7a. The mass transport in the Y-direction was limited to diffusion, resulting in a boundary on the membrane surface. As shown in Figure 7a, water velocity decreased toward the reactor peripherals. The decline in velocity and boundary layer constraints may prevent naproxen from reaching the membrane’s active site, limiting the process’s efficiency. However, by optimizing stirring speed, convective flow mass transport can be introduced into the photocatalytic membrane reactor. Convective current increases the chances of collision between the naproxen and active sites of the membrane; this reduces boundary layer thickness, making the photocatalytic membrane reactor less dependent on diffusion as a mass transfer process.

In the membrane separation unit, the simulation was carried out following the fluid channel passing through the membrane surface (Figure 7b). The fluid flow profile demonstrated a crossflow mechanism. Near the membrane, the velocity assumes a linear profile in the vertical (Y) direction to facilitate permeation. The fluid channel entered the membrane separation unit along the X-direction, and upon permeating through the membrane, the fluid channel flowed along the Y-direction (Figure 7b). In the X-direction, a uniform flow was observed; however, upon contact with the membrane surface, turbulences with different velocities were obtained. The turbulences were attributed to rapid changes in the flow direction. Overall, velocity dropped as the water permeated through the membrane. The change in flow direction induced convection flow, making it the dominant mass transport process for naproxen. The convective flow circumvents mass transfer constraints and eliminates the concentration differential in the boundary layer by transferring naproxen to the membrane surface from the bulk solution. However, in terms of rejection, the degree of velocity directly impacted the process performance, where higher velocities may result in lower rejections, as seen in the experimental data (Section 4.6.5). At higher velocities, the magnitude of forcing naproxen through membrane pores may overpower the rejection properties of the membrane, leading to low rejections.

### 4.5. Effects of Light Distribution

**Figure 8:**
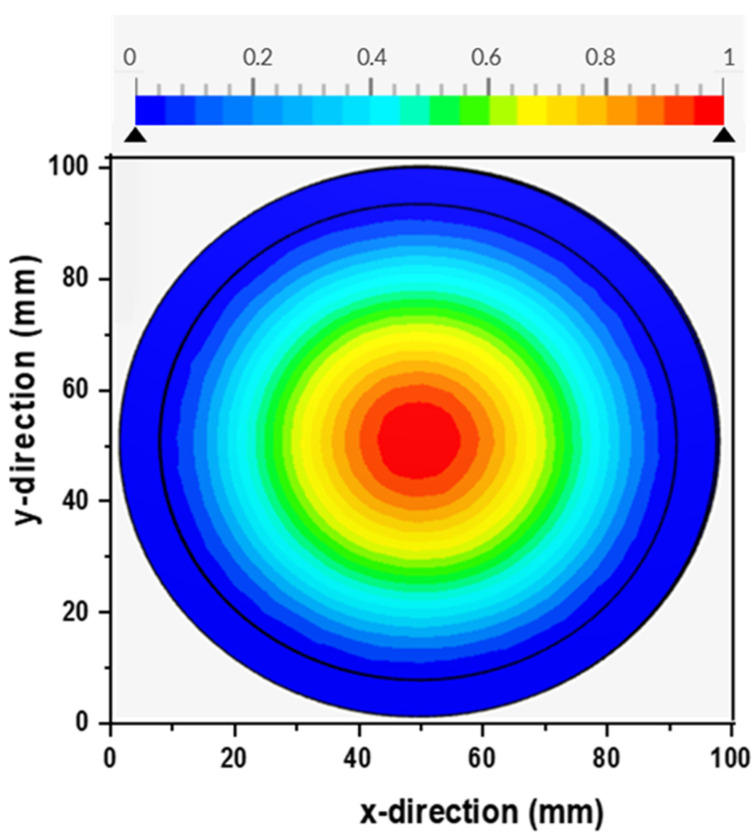
Incident irradiation distribution over a flat-sheet photocatalytic membrane (lamp position at 150 mm from the membrane surface).

The photonic efficiency in photocatalytic membranes is highly dependent on the dispersion of light over the membrane. Figure 8 depicts the simulated homogeneity of the light on the surface of a flat-sheet photocatalytic membrane. The light source was positioned 150 mm from the reactor’s top surface. It is known that the irradiation distribution profiles can be utilized to improve the efficiency of the process by spotting dimly illuminated regions in the reactor [29]. Using Equation (9), the incident radiation uniformity index was derived to assess light distribution on the membrane surface. The incident radiation uniformity index measures the uniformity of a changing field over a surface and is based on local fluctuations in relation to the mean value of the irradiated area. An index value of 1.0 denotes the highest uniformity. In this study, a homogeneity index for incident radiation of 0.56 was attained. The index value 0.56 is comparable to the results obtained in previous reports [12]. Casado et al. [12] illustrated that a higher degree of uniform light distribution can be obtained when smaller areas are employed.

**Figure 9:**
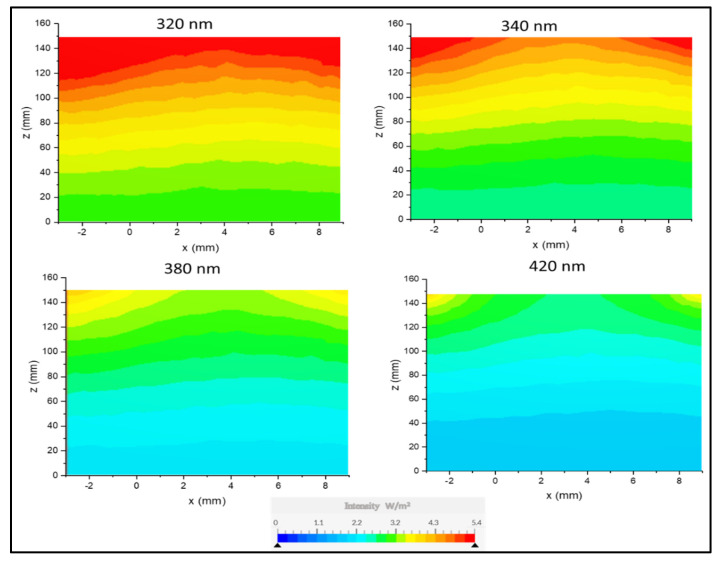
Axial (x, z) contour plots of the irradiation distribution inside the reactor from a light source positioned at 150 mm to the surface of the membrane at the bottom.

Given the obtained irradiation distribution value (0.56) on the membrane surface along the *X* and *Y* plane, it was necessary to predict how irradiation was dispersed throughout the reactor as this might have a significant impact on the photonic efficacy of the process. The reactor was positioned vertically to ensure that the beams irradiate the reactor at the zenith angle. The radiation transport equation (RTE) was solved at four different light wavelengths, and the distribution of the irradiation intensities was simulated, as shown in Figure 9.

The profiles (Figure 9) illustrated that the intensity of the light declined towards the surface of the membrane. Along the radial coordinate, the light intensity was generally homogeneous, except for a small region at the top of the reactor. The decline in intensity was attributed to the impact of the redistribution of photons and distance from the source. These findings correspond to another study in the literature [9]. The amount of irradiation reaching the membrane surface generally influences the rate of photogeneration of reactive species; hence, high intensities were anticipated to increase degradation performance. This was confirmed via experimental data in Section 4.6.6. Examination of Figure 9 also showed that the membrane had a stronger absorption of specific wavelengths below 380, as indicated by the lime green color on the scale bar. The measurement at a wavelength of 380 nm resulted in an index number of 0.56, motivating the need to investigate the system behavior further under different optical conditions and optimize performance for specific wavelengths. The intensity produced by the light source is influenced by the wavelength, which, in turn, affects the absorption of light on the membrane surface.

### 4.6. Experimental Performance Studies

#### 4.6.1. UV-Vis Spectra of Photolysis and Photocatalytic Degradation of Naproxen in the Batch Reactor

**Figure 10:**
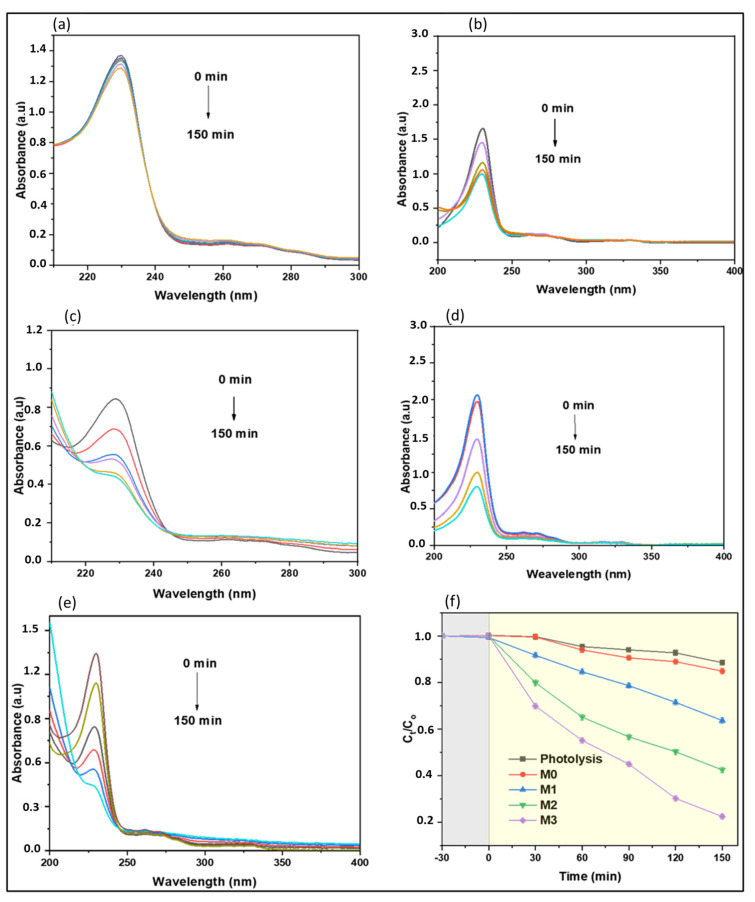
Variation of the UV-Vis spectra of naproxen over time in a batch reactor by (**a**) photolysis, and photocatalysis degradation using CoFe_2_O_4_-PES membranes; (**b**) M0—pristine; (**c**) M1—0.2 wt.% CoFe_2_O_4_; (**d**) M2—0.5 wt.% CoFe_2_O_4_; and (**e**) M3—1 wt.% CoFe_2_O_4_; (**f**) effect of time on the degradation of naproxen (grey zone incidates experiment without irradiation and yellow zone indicates experiment under irradiation).

The photocatalytic degradation performance of the prepared membranes was evaluated with a naproxen contaminant in spiked real-water samples. Figure 10 illustrates the UV absorption spectra of the reaction aliquots at various time intervals of photolysis and photocatalytic degradation studies. The major absorption band for naproxen was identified at 230 nm. Naproxen removal may occur via photosensitization and photocatalytic degradation in the presence of light; hence, the contribution of photosensitization to the overall degradation was investigated using photolysis. Further, the addition of photocatalytic CoFe_2_O_4_-PES membranes as photocatalysts resulted in faster degradation of naproxen, compared with the photolysis, and significant removal of naproxen and its intermediates seemed to have occurred (Figure 10b–e).

#### 4.6.2. Effects of Time on Naproxen Degradation Performance

Simulation studies indicated the role of light distribution and intensities on the accessibility of the irradiation to the photocatalytic membrane and the role of mass transfer of the solute to the photocatalyst on degradation performance. However, photocatalysis is also linked to parameters such as irradiation time. In line with improving degradation performance, the influence of irradiation time on degradation performance was investigated. The reactive mixture was irradiated for 150 min (Figure 10f) with other parameters, such as naproxen concentration, mixing speed, and pH, held constant. The degradation performance of all membranes improved with time, with M3 reducing the initial concentration to 0.38, whereas M0, M1, and M2 achieved 0.88, 0.76, and 0.61, respectively. An increase in irradiation time may have pre-conditioned the membrane samples, giving sufficient time for nanoparticles covered by the PES matrix to be accessible to light, hence generating sufficient reactive radicals for the degradation of naproxen. In addition to being time-dependent, degrading naproxen performance was also observed to increase significantly with CoFe_2_O_4_ loading in membranes over time (Figure 10f). From data shown in Figure 10, M3 performed better than other membranes, hence 1 wt.% CoFe_2_O_4_ loading was chosen as the optimum membrane loading for this study. Based on this, M3 was used for further investigation.

#### 4.6.3. Effects of pH on the Degradation of Naproxen

**Figure 11:**
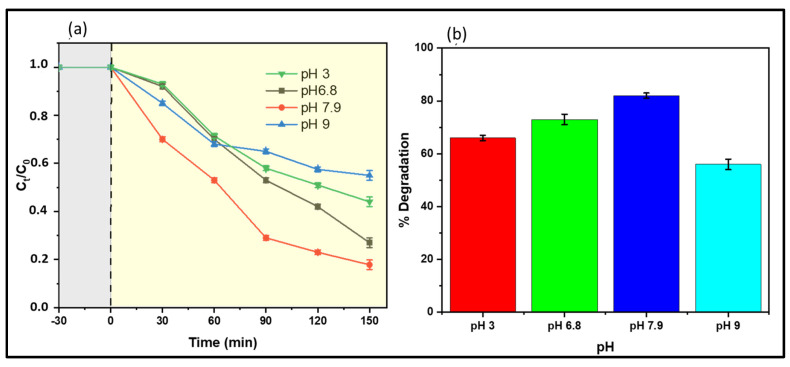
(**a**) Change in concentration with time at different pH (grey zone incidates experiment without irradiation and yellow zone indicates experiment under irradiation) and (**b**) degradation performance at different pH. Role of pH during naproxen degradation using a 300 rpm mixing rate, naproxen concentration of 5 mg/L of water samples collected before chlorination from a drinking water treatment plant, and M3 −1 wt.% CoFe_2_O_4_.

pH plays a critical role in the photocatalytic degradation process, as it affects the speciation of photocatalysts and targeted contaminants. Figure 11 illustrates the effect of pH on naproxen degradation. The results infer that the heterogeneous system can remove naproxen over a broad range of pH from 3.0 to 9.0, which covers the pH range of most real drinking water and wastewater. Lower degradation performances were detected at strongly alkaline (pH 9) and acidic (pH 3) conditions. Under acidic conditions, less degradation profile (Figure 11a) was ascribed to adding HCl solution during pH adjustments. The Cl^−^ ions from HCl may have reacted with ^•^OH radicals and produced inorganic radicals (such as ClOH^−•^). These ClOH^−•^ anion radicals generally have lower reactivity than hydroxyl radicals. As a result, a competition between the naproxen and Cl^−^ ions for ^•^OH radicals occurred, inhibiting naproxen degradation. On the other hand, Gong et al. [30] reported a positive effect that resulted from the dissociation of ClOH^−•^ to Cl^•^; this was not the case in this study. Excess chloride ions may have reacted with the generated Cl^•^ to form Cl_2_^−•^ (Equation (19)) with low reactivity.

(18) $$Cl^{-}+OH^{•}\;↔\;ClOH^{-•}\;\to H^{+}+\;Cl^{•}+H_{2}O\;$$

(19) $$Cl^{•}+Cl^{-}\;\to \;Cl_{2}^{-•}$$

The performance increased with an increase in pH values from acidic to almost neutral (7.9); thereafter, the performance decreased in the alkaline region to pH 9 (Figure 11b). In strong alkaline conditions (pH 9), the degradation performance declined to 55%, which was ascribed to the production of potential ^•^OH scavengers such as OH^−^. Hydroxyl ion (OH^−^) reacted with the ^•^OH radical to produce O^−•^, characterized by lower oxidative capability.

#### 4.6.4. Effects of a Water Matrix on Remediation of Naproxen

**Figure 12:**
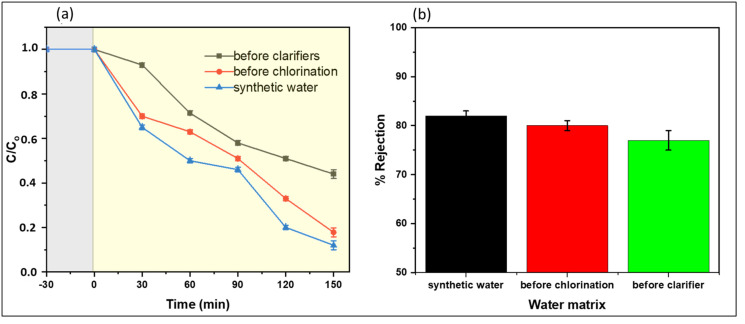
Impacts of the water matrix on remediation of water containing naproxen (**a**) degradation (grey zone incidates experiment without irradiation and yellow zone indicates experiment under irradiation) and (**b**) rejection of 5 mg/L naproxen using M3 (1 wt.% CoFe_2_O_4_) membrane.

The effect of the water matrix on the degradation of naproxen is shown in Figure 12a. From the obtained data, naproxen concentration decreased up to 0.12 when synthetic water was used. For water sampled before clarification, 0.46 naproxen concentration was left after 150 min of degradation. The decline in performance was ascribed to the presence of other natural organic matter (NOM) in addition to naproxen. These NOMs could have had scavenger effects of reactive radicals such as HO^•^, resulting in competitively consumed radicals.

Furthermore, water samples collected before the clarifier had the highest turbidity (22.5 ± 2.6 (Table S1)). High turbidity acted as an inner irradiation filter, hence minimizing the accessibility of irradiation to the membrane’s active sites, leading to insufficient generation of reactive species. The performance of water samples collected before clarification was attributed to low turbidity of 6.2 ± 0.02 and low concentration of NOM. Regarding rejection, no significant effect of the water matrix was observed on performance towards naproxen rejection (Figure 12b). Rejection depends on the target pollutant’s properties and the membrane’s characteristics, including pore size and electrostatic and non-electrostatic interactions. In this work, using CoFe_2_O_4_-PES membranes demonstrated physiochemical properties that facilitated the rejection of naproxen through multiple mechanisms. Adding CoFe_2_O_4_ nanoparticles to the membranes resulted in the formation of a dense and thick active layer (Figure S1), effectively reducing the pore size. This decrease in the membrane’s molecular weight cut-off (MWCO) limited the passage of naproxen, leading to higher removal through size exclusion or sieving effects.

Naproxen, which has a Log K_ow_ value of 2.986, exhibits slight hydrophobicity and carries a negative charge at neutral pH [19]. The hydrophilic nature (Figure S5) of the CoFe_2_O_4_-PES membrane reduced the hydrophobic interactions between the membrane and naproxen. The hydrophilic nature of the membranes led to the formation of a hydration layer on their surfaces. This hydration layer increased the repulsive interactions between the membranes and naproxen, resulting in higher rejection rates. Consequently, the naproxen rejection increased, consistent with previous findings reported in the literature [31]. Further, these repulsive interactions between the membrane and naproxen also hindered the partitioning of naproxen through the membranes. This resistance further contributed to the overall rejection of naproxen.

Charge interactions are also known to play a role in the rejection process. The negatively charged naproxen molecules were repelled by the negatively charged membranes (Table 1) owing to the deprotonation of functional groups on the membrane surface, leading to the observed rejection. Within the pH range of 5 to 8, naproxen carries a partial negative charge, contributing to its rejection on the membrane surface.

Furthermore, the rejection phenomenon may be attributable to specific interactions between the membrane and naproxen, such as van der Waals forces, hydrogen bonding, and dielectric effects [32]. These interactions can form a hydration layer on the membrane surfaces, increasing the repulsive hydration interactions between the membranes and naproxen. This leads to higher rejection rates.

A notable decline in flux during the operation was observed from water samples collected before clarification (3.65 ± 0.2 L.m^−2^.h^−1^), whereas for synthetic water and water samples collected before chlorination, the flux slightly decreased (8.57 ± 0.21 and 8.47 ± 0.2 L.m^−2^.h^−1^, respectively) within 15 min of operation (Table 1).

The decline in water flux was due to the formation of a cake layer on the membrane surface, which decreased the permeability of the membranes. The data showed that water with high turbidity required pre-treatment processes before applying photocatalytic degradation and membrane separation.

#### 4.6.5. Effects of Mixing Velocity and Flow Rate on the Removal of Naproxen

**Figure 13:**
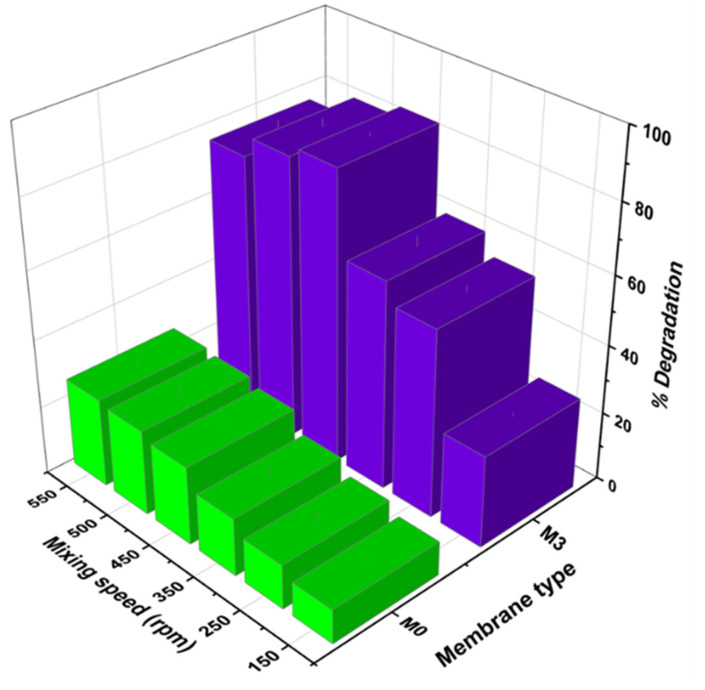
Influence of mixing velocity on the removal of naproxen in water samples collected before chlorination at pH 6.8 and naproxen concentration of 5 mg/L.

The influence of different mixing velocities on naproxen degradation is illustrated in Figure 13, where pristine membrane (M0) was used as the control experiment. The increment in mixing speed improved degradation performance from 44% (150 rpm) up to 84% (450 rpm) for M3 (Figure 13). A similar trend was observed for M0. M0’s performance increased from 12 to 18%. The increase in performance was attributed to the kinetic energy gained by the reactive species and naproxen. This improved the intensive interfacial contact between reactive species and facilitated optimal mass transfer. The chances of successful collision between reactive radicals and naproxen molecules in the photocatalytic process improved with the magnitude of speed. Visan et al. reported similar findings [33]. However, this study noted that after 450 rpm, there was a slight decrease in performance. This decline could emanate from the ineffective collision of reactive species due to rapid motion beyond optimum effect. These results confirm the findings obtained with simulation studies (Section 4.3). Although a well-established correlation exists between increased mixing and higher convective mass transfer, most research in this field has focused primarily on slurry photocatalytic degradation systems. Comparatively, fewer studies have been conducted on immobilized systems, particularly batch reactor systems with flat sheet membranes and liquid systems. The primary objective of our study was to investigate whether convective mass transfer can effectively facilitate pollutant interaction with the active sites in an immobilized system without filtration. By examining this specific configuration, we aimed to bridge the gap in the existing literature and shed light on the effectiveness of convective mass transfer in such systems.

**Figure 14:**
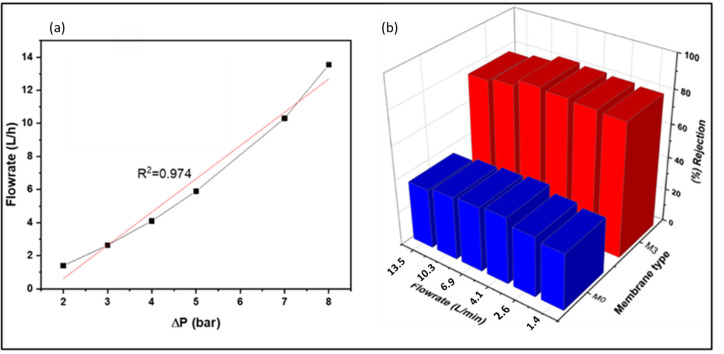
(**a**) Correlation of flow rate and pressure using Darcy’s law. (**b**) Effect of flow rate on the removal of naproxen.

Figure 14 presents the influence of flow rate on naproxen rejection. The relationship between pressure and the flow rate was computed using Darcy’s law, Equation (20).

(20) $$Q=\frac{A\times D\times \Delta P}{L}$$

where Q is the volumetric flow rate, A is the cross-sectional area of the membrane, D is the permeability coefficient, ΔP is the pressure difference across the membrane, and L is the length of the membrane. The Darcy model illustrated a relationship with R^2^ = 0.974 (Figure 14a). Indicating ease of calibration between pressure and flow rate in systems that employ nanocomposite membranes; hence, one property (pressure or flow rate) was investigated instead of both. To understand how flow rate affects the removal performance of naproxen, flow rates in the range of 1.4 to 14 were investigated.

As shown in Figure 14b, M3′s rejection slightly increased by 3% up to 6.5 L/min and declined by 5% at 13.5 L/min flowrate. The observed rejections trend can be explained by the shielding effect phenomenon. As explained in Section 4.4 (simulations studies), low flow rates can result in the build-up of naproxen at the membrane surface, as the boundary layer constantly transfers naproxen from the bulk solution to the membrane surface. This could result in concentration polarization. However, the crossflow mechanism is more dominant at a high flow rate. The feed flow rate has enough power to sweep off the layer building up on membrane surfaces.

On the other hand, a slight decline in rejection performance was observed after a flow rate of 6.5 L/m. The slight decline in rejection performance was attributed to high feed flow rates, which may have overpowered the rejection properties of the membranes. This resulted in the passage of naproxen through the membrane pores, and similar trends were obtained for M0.

#### 4.6.6. Effects of Light Intensity on the Degradation of Naproxen

**Figure 15:**
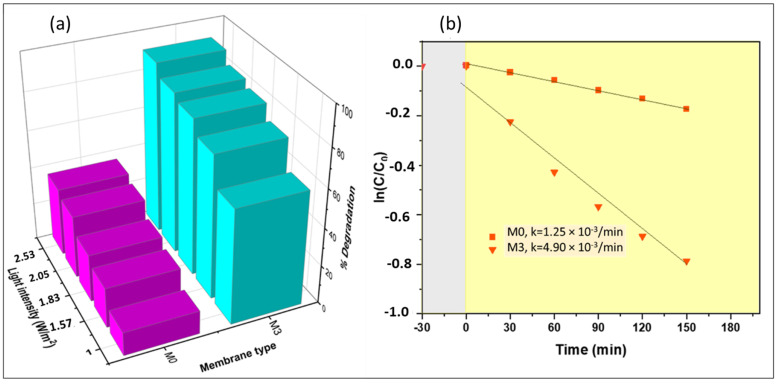
(**a**) Effects of light intensity on the naproxen degradation process and (**b**) the naproxen degradation kinetics profile (grey zone incidates experiment without irradiation and yellow zone indicates experiment under irradiation).

A Lux meter (GM 1020, China) was used to measure variations in light intensity. Conversion of Lux to watts was achieved using Equation (21).

(21) $$P=E_{V\;\left(lux\right)}\times A/\eta$$

where P is the power in watts, $E_{V}$ is the illuminance in lux, *A* is the irradiation area, and η is the luminous efficacy. Figure 15 depicts the influence of light intensity on the degradation of naproxen. Degradation performance for M3 was observed to improve with light intensity (from 60 to 91%). An increment in light intensity increased the supply of photon energy, which enhanced photoexcitation of the active sites of the membrane, and more reactive radicals were generated. It is reasonable to think that, at higher light intensities, the light irradiates deeper into the membrane, thus activating more CoFe_2_O_4_ nanoparticles embedded in the membrane. These results agreed with CFD radiation simulation results (Section 4.5). An increase in light intensity resulted in more photons being absorbed by the membrane’s active sites. Similar trends were observed for M0. The membrane M0 served as the control in our study and was also used to demonstrate the contribution of photosensitization as the light intensity was increased. As the intensity of light increases, the photosensitization of naproxen also increases. However, for membrane M3, despite the contribution from photosensitization, the dominant mechanism for pollutant removal was photocatalytic degradation.

#### 4.6.7. Kinetics Studies for the Degradation of Naproxen

Optimum conditions obtained from the investigated parameters influential to naproxen degradation (mixing speed, intensity, and others) were used to compute the kinetics of naproxen degradation in the batch reactor. However, the pH value of real water containing naproxen is generally known to be neutral. This is why, in this study, although the degradation showed high removal values at pH 7.9, approximately 8, the experiments were carried out at pH 6.8, approximately 7, neutral values. The obtained reaction kinetics obeyed the apparent first-order kinetic model. The rate constant for M3 was 4.90 × 10^−2^ min^−1^ (at R^2^ 0.9739), showing that process optimization played a role in improving the accessibility of the irradiation to photocatalytic nanoparticles and the rate of diffusion and mass transfer of the solute to the photocatalyst surface. Further, the reaction conversion (x) of the batch reactor system was calculated using Equation (22).

(22) $$x=\frac{moles\;of\;naproxen\;reacted\;at\;time\;t}{mole\;of\;naproxen\;fed\;to\;the\;system}$$

A conversion of 0.91 was obtained after 150 min of operation using M3. This indicated that the high degradation performance of naproxen was achieved.

#### 4.6.8. Application of IPMS on the Removal of Naproxen

IPMS was operated under optimum conditions obtained from previous activities in which photocatalysis and rejection were operated as stand-alone. A feed solution of 5 mg/L of naproxen was initially degraded, and the resultant solution was then conveyed to the rejection unit. Table 2 shows the change in water quality after treatment with the IMPS system.

**Figure 16:**
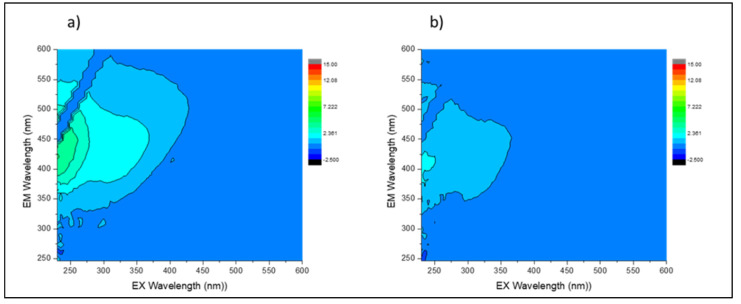
FEEM analysis for (**a**) untreated and (**b**) treated water samples collected before chlorination using IPMS.

The intensity of water quality parameters such as TDS, turbidity, TOC, and electrical conductivity was reduced to acceptable levels for potable water according to SANS 241-1:2015 drinking water specifications. Figure 16 shows a decrease in NOM after treatment with IPMS. Fluorescent NOM in the water samples was analyzed using fluorescence excitation–emission matrix (FEEM) spectroscopy. Parallel factor analysis was used to identify the NOM present in the water samples from the FEEM data. Three typical components were identified: (i) at excitation/emission (Ex/Em) = 270/470 nm, which was related to the humic-like matters; (ii) at Ex/Em = 350/480 nm related to fulvic acids; and (iii) at Ex/Em 275/340 related to tryptophan or the protein-like substances derived from biological production in surface water [34]. The spectra of the three typical components described above are displayed in Figure 16a,b representing two statuses of water, i.e., before treatment and after treatment. Marais et al [35] have reported similar FEEM images data for raw water (untreated water) samples.

Based on the obtained conversion (Section 4.6.7) and rejection (Section 4.6.5), the overall change in concentration of naproxen was computed following relations adapted from Levenspiel [36] (Equation (23)).

(23) $$C_{f}=C_{i}-\partial C_{degradation}-\partial C_{rejection}$$

where $C_{f}$ is the naproxen permeate concentration, $\partial C_{degradation}$ is the concentration of naproxen resulting from degradation, and $\partial C_{rejection}$ is the concentration of naproxen changed due to rejection. The IPMS process achieved 99% naproxen removal efficacy, whereas stand-alone processes (photocatalysis and rejection) obtained up to 83%. Therefore, it can be affirmed that the integrated system performs better than the stand-alone process in this study.

The efficiency of the photocatalytic process is determined by photon transfer and mass energy efficiency, throughput, and photocatalytic activity transfer. Space–time yield (STY) and photocatalytic space–time yield (PSTY) are the models that can be used to provide a measure of performance in terms of throughput, energy use, and photocatalytic activity. In this work, STY was defined as the amount of contaminant degraded per reactor volume per time, as expressed by Equation (24). STY essentially reflects the effect of reaction mass and photon transfer rates on reactor productivity [37]. The obtained STY and PSTY are summarized in Table 3, which was 1.23 × 10^12^ mol/cm^2^.s.

(24) $$STY=\frac{C_{0}\times \left(1-e^{-kt}\right)\times V_{r}}{A_{membrane}\times t}\times \frac{1}{M_{naproxen}}$$

where is $C_{0}$ is the naproxen initial concentration (g/cm^3^), k is the apparent rate constant (s^−1^), t is the reaction time (s), $V_{r}$ is the volume of the $V_{r}$ (cm^3^), $A_{membrane}$ is the membrane surface area (cm^2^), and $M_{naproxen}$ is the molar mass of naproxen (g/mol). PSTY is simply the ratio of STY to the power intensity received by the reactor. Thus, PSTY considers light utilization efficiency [36], which was obtained using Equation (25), where PI is light intensity. At 2.5 W/m^2^, PSTY was 4.93 × 10^11^ mol/W.s.

(25) $$PSTY=\frac{STY}{PI}$$

Table 4 provides a precise comparison of data from the open literature and the results of this study. The comparison reveals that the IPMS presented in this work is highly competitive compared to other reactor systems in terms of STY and PSTY.

## 5. Conclusions

A CFD-assisted process optimization of an integrated photocatalytic membrane system for water treatment was studied. The role of irradiation distribution and light intensity on the performance of a CoFe_2_O_4_-PES membranes-based photocatalytic batch reactor was investigated. This exploration is crucial for optimizing the design and operation of photocatalytic reactors, and it allowed us to identify key factors that influence the overall performance. Furthermore, the role of mass transfer and fluid dynamics on the performance of photocatalytic membrane systems without a flow-through mechanism was evaluated. The performance of photocatalytic degradation, rejection process, and integrated system were studied. Parameters essential to naproxen removal performance were investigated and optimized, including pH, water matrix, flow rate, mixing velocities, and light intensity. Extreme pH conditions (pH 3 and 9) showed a negative effect on naproxen removal performance, whereas flow rate, mixing velocities, and light intensity increased performance until optimum conditions were reached. Under optimum conditions, 4.93 × 10^11^ mol/W.s naproxen removal was achieved. The degradation efficiency was attributed to high mass transfer and effective illumination, which increased the probability of naproxen and reactive radicles colliding. The IPMS performed better (99%) than photocatalytic degradation (81%) and rejections (83%) as stand-alone. Therefore, IPMS is suitable for the remediation of naproxen from drinking water, and reactor designs based on such a system are a necessity. Further, the obtained experimental results were in agreement with the simulation results from CFD. The photocatalytic effectiveness and IMPS energy efficiency were also assessed. The data showed that IPMS can perform well with space–time yield (STY) (1.23 mol/cm^2^.s 10^11^) and photocatalytic space–time yield (PSTY) (4.39 mol/W.s 10^11^). This developed IPMS can be competitive regarding energy intensiveness and throughput capacity for water treatment.

## Figures and Tables

**Table 1:** Water flux and zeta potentials for the M3 (1 wt% CoFe_2_O_4_-PES) membrane.

Flux (L.m^−2^.h^−1^)				Zeta Potential	
Time (min)	Synthetic Water	Before Chlorination	Before Clarifiers	pH	mV
15	8.47 ± 0.2	8.47 ± 0.2	3.65 ± 0.2	5	−18.8 ± 1
30	8.02 ± 0.3	7.98 ± 0.2	1.23 ± 0.4	6.8	−25.5 ± 2.2
45	7.85 ± 0.3	7.65 ± 0.2	0.25 ± 0.05	8.3	−32.9 ± 2.2

**Table 2:** Change in water quality parameters of the collected water from the drinking water treatment plant after IPMS treatment.

Water Parameter	Before Treatment	After IPMS Treatment
pH at 25 °C	6.8 ± 0.6	6.8 ± 0.23
TDS (ppm)	63.8 ± 4.2	28.9 ± 1.2
Turbidity (NTU)	6.2 ± 0.02	0 ± 0.6
TOC (mg C.L^−1^)	10.4 ± 1.2	4.3 ± 0.4
Electrical conductivity (mS/m) 25 °C	119 ± 4	67.6 ± 1.5
Naproxen (mg/L)	5 ± 0.01	0.14 ± 0.01

**Table 3:** IPMS efficacy in terms of STY, PSTY, conversion, and rejection.

Parameters	Degradation	Rejection
Illuminance system	Solar simulator	-
Light intensity (w/m^2^)	2.5	-
Residence time (m)	150	-
Flow rate (L/m)	-	6.8
Active membrane area (m^2^)	0.0028	0.0028
STY (mol/cm^2^.s)	$1.23\times 10^{12}$	-
PSTY (mol/W.s)	$4.93\times 10^{11}$	-

**Table 4:** Comparison of STY and PSTY for different reactors.

Reactor	Performance (%)	$\mathrm{STY}\;\mathrm{mol}/{\mathrm{cm}}^{2}.s\;(10^{11}$)	$\mathrm{PSTY}\;\mathrm{mol}/W.s\;(10^{11})$	Ref.
Micro-channel reactor	64	0.00023	0.000828	[37]
Flat-plate solar photocatalytic reactor	99	0.171	0.612	[38]
Photocatalytic membrane reactor	80	1.6	0.5	[12]
IPMS	99	1.23	4.39	Current work

## Supplementary Materials

The following supporting information can be downloaded at: https://www.mdpi.com/article/10.3390/membranes13100827/s1, Figure S1. Physical (surface (a–d) and cross-sectional micrographs (e–h)) of pris-tine and nanoparticle modified PES membranes: M0—pristine (a & e); M1—0.2% CoFe_2_O_4_ (b & f); M2—0.5% CoFe_2_O_4_ (c & g) and M4—1% CoFe_2_O_4_ (d & h); Figure S2. Optical properties of CoFe_2_O_4_ nanoparticles and CoFe_2_O_4_-PES membranes: (a) UV Visible spectrum of CoFe_2_O_4_ particles (b) Tauc plot showing the band gap of the magnetite particles (c) UV-Visible spectrum of CoFe_2_O_4_-PES membranes; Figure S3. Definition of simulated rays’ number; Figure S4. Chemical composition (EDS) for the fabricated membranes (M0 (a), M1 (b), M2 (c), and M3 (d)): M0 with 0% CoFe_2_O_4_ nanoparticles; M1 with 0.2% CoFe_2_O_4_ nanoparticles; M2 with 0.5% CoFe_2_O_4_ nanoparticles and M4 with 1% CoFe_2_O_4_ nanoparticles; Figure S5. Water contact angle for pristine and CoFe_2_O_4_ modified membranes; M1 with 0.2% CoFe_2_O_4_ nanoparticles; M2 with 0.5% CoFe_2_O_4_ nanoparticles and M4 with 1% CoFe_2_O_4_ nanoparticles; Figure S6. Water permeability of the prepared membranes; M0 pristine M1 with 0.2% CoFe_2_O_4_ nanoparticles; M2 with 0.5% CoFe_2_O_4_ nanoparticles and M4 with 1% CoFe_2_O_4_ nanoparticles; Figure S7. FTIR analysis of prepared CoFe_2_O_4_-PES membranes; Table S1. Water quality parameters of the collected water samples. References [35,39,40,41,42,43,44,45] are cited in Supplementary Materials file.
