# Supplementary material for: CFD-Assisted Process Optimization of an Integrated Photocatalytic Membrane System for Water Treatment
Source: Membranes (Basel). 2023 Oct 9;13(10):827. doi: 10.3390/membranes13100827 (PMC10608198; doi:10.3390/membranes13100827)
Supplement: Supplementary file 1 [file membranes-13-00827-s001.zip › membranes-2596381-supplementary.pdf]

Supplementary material

CFD-Assisted Process Optimization of an Integrated Photocatalytic Membrane System for Water Treatment

Vimbainashe Mercy Chakachaka, Charmaine Sesethu Tshangana, Bhekie Brilliance Mamba and Adolph Anga Muleja *

Institute for Nanotechnology and Water Sustainability, College of Science, Engineering and Technology, University of South Africa, Florida Science Campus, Johannesburg 1709, South Africa
* Correspondence: mulejaa@unisa.ac.za

S1. Real water sample analysis

A summary of the real water parameters is given in **Table S1**. Samples were collected from two sampling points, before clarifiers (A) and before chlorination (B) to achieve the objectives of this chapter. Further, the objective of the study was to investigate the potential of using IPMS as a secondary treatment process, hence the focus was on the treatment of feed water collected before chlorination, unless stated otherwise. Based on SANS 241-1:2015 drinking water specifications, both sampling point A and B did not meet all the standard requirements for potable water [1].

Table S1. Water quality parameters of the collected water samples.

Water parameter	Before clarifiers	Before chlorination
pH at 25℃	6.9 $\pm$ 0.6	6.8 $\pm$ 0.6
TDS (ppm)	124 $\pm$ 4.9	63.8 $\pm$ 4.2
Turbidity (NTU)	22.5 $\pm$ 2.6	6.2 $\pm$ 0.08
TOC (mg C.L^{-1})	26.3 $\pm$ 1.2	10.4 $\pm$ 1.2
Electrical conductivity (mS/m) 25℃	210 $\pm$ 4.3	119 $\pm$ 4

S2. Fabrication and characterization of membranes

The membranes were prepared using $CoFe_2O_4$ nanoparticles, polyether sulfone (PES), and N-Methyl-2-pyrrolidone (NMP) *via* the phase inversion method as described in our previous work [2]. Characterization of the developed membranes is also discussed in detail in that previous study [2]. The characteristics linked to this work such as optical properties, surface, and cross-sectional morphology, contact angles, and zeta potential are provided in this work.

S3. Surface and cross-sectional analysis of the prepared samples

Surface and cross-sectional SEM micrographs of the pristine and $CoFe_2O_4$-PES membranes are displayed in (**Figure S1**). The images showed morphological changes with the addition of $CoFe_2O_4$ nanoparticles. The membrane samples (**Figures S1a and b)** revealed relatively regular surfaces. M1 had a regular surface due to the low (0.2 wt. %) nanoparticles loading (**Figure S1b**). However, Figures **S1c** and **S1d** showed agglomerate-like on the surfaces of the membranes compared to that of the pristine PES membrane (**Figure S1a**) with **Figure S1d** exhibiting rougher surface as it was loaded with higher (1 wt. %) nanoparticles. This suggested that the $CoFe_2O_4$ nanoparticles might have effectively penetrated PES polymer chains, and nanoparticles were well dispersed in the membrane.

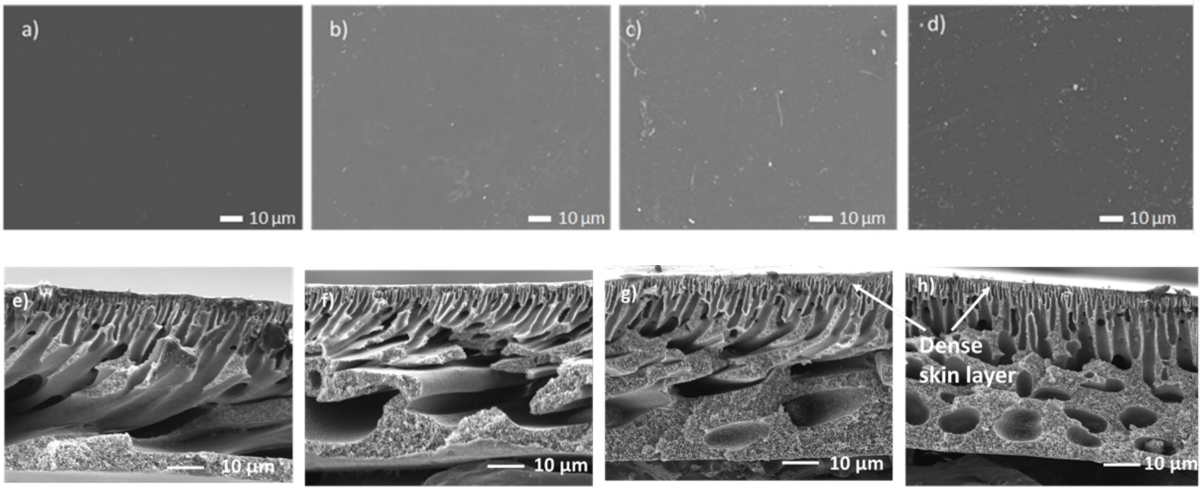

Figure S1. Physical (surface (a-d) and cross-sectional micrographs (e-h)) of pristine and nanoparticle modified PES membranes: M0 - pristine (a &e); M1 – 0.2% $CoFe_2O_4$ (b & f); M2 – 0.5% $CoFe_2O_4$ (c & g) and M4 – 1% $CoFe_2O_4$ (d & h).

All the fabricated membranes were made of an asymmetric membrane structure consisting of a thick top layer and a finger-like microporous structured porous sub-layer (**Figure S1e, f, g,** and **h**). From the micrographs, an observation was made that the finger-like structures increased with $CoFe_2O_4$ nanoparticles loading. $CoFe_2O_4$ nanoparticles increased the hydrophilicity of the casting solution, which facilitated quick interchange between the NMP solvent and water in the coagulation bath, increasing the population of pores [3]. However, the addition of the $CoFe_2O_4$ nanoparticles at loadings more than 0.5 wt. % (Figure 8d) led to the formation of denser skin layers with increased thickness.

S4. UV vis spectra for $CoFe_2O_4$ nanoparticles

A UV-Vis spectrum shows that $CoFe_2O_4$ nanoparticles had adsorption bands from 300 to 800 cm^{-1} showing that the nanoparticles were sensitive in the visible region (**Figure S2a**). Using the Tauc plot, the band gap energy for $CoFe_2O_4$ nanoparticles was 1.85 eV (**Figure S2b**). By integrating polyethersulfone (PES) and $CoFe_2O_4$ nanoparticles, the bandgap of the resulting mixed matrix membrane was modified, and intra-band gap states were introduced. These modifications were expected to reduce the recombination of electron-hole pairs, enhance light absorption, promote electron delocalization, and improve charge carrier mobility. The UV-vis results (Figure S2c) obtained further supported these modifications, demonstrating the light absorbance properties of the synthesized membranes.

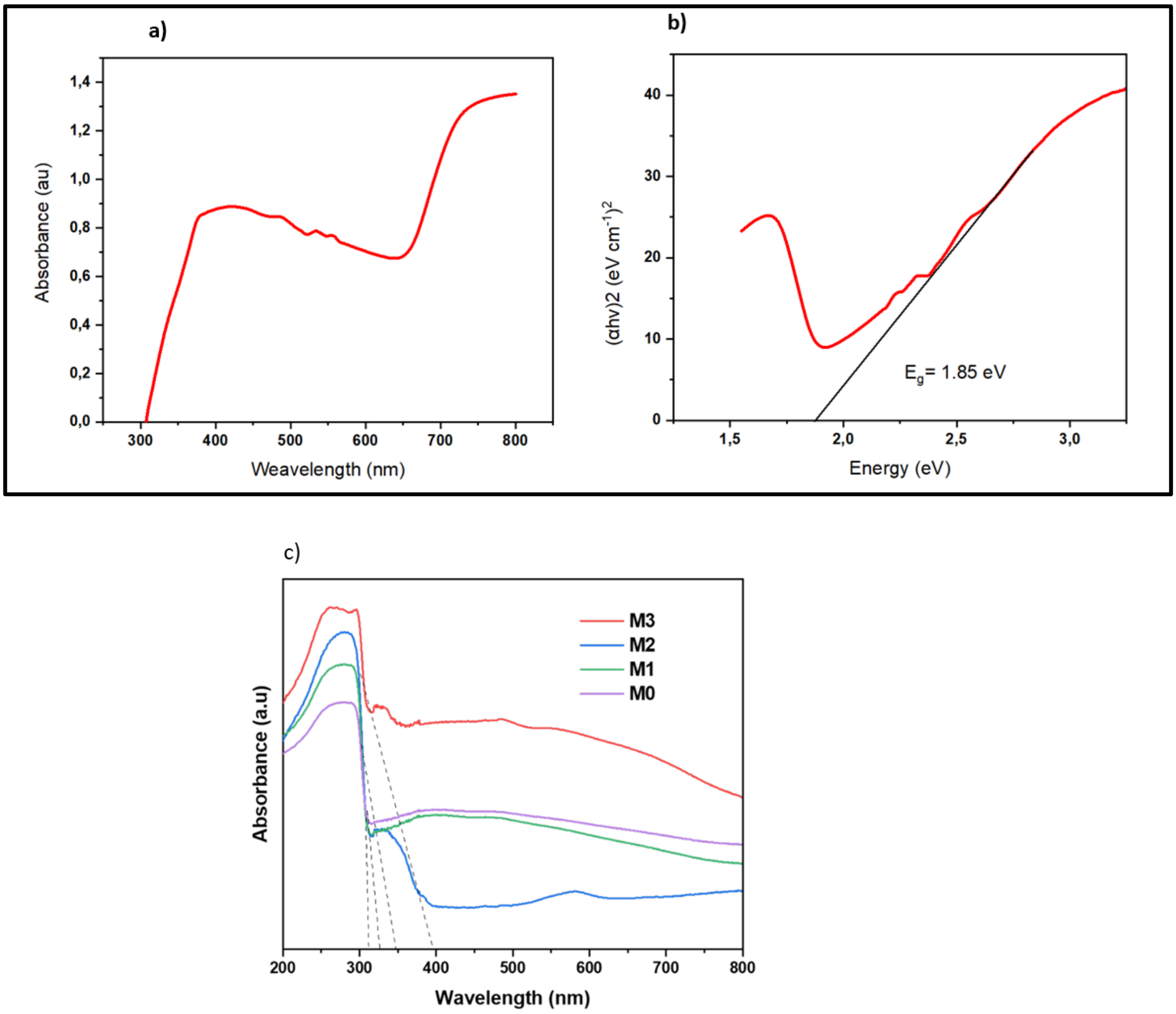

Figure S2. Optical properties of $CoFe_2O_4$ nanoparticles and $CoFe_2O_4$-PES membranes: a) UV Visible spectrum of $CoFe_2O_4$ particles b) Tauc plot showing the band gap of the magnetite particles c) UV-Visible spectrum of $CoFe_2O_4$-PES membranes.

S5. Ray tracing

The non-sequential ray tracing algorithm was conducted and solved based on the Monte Carlo method and in the principles of ray optics. The number of traced rays defines the precision of the results. Therefore, a test to determine the ideal number of traced rays was performed. Six simulations were performed for a total of 10^5, $6{\cdot}10^5$, 10^6, $6{\cdot}10^6$, 10^7 and $6{\cdot}10^7$ traced rays.

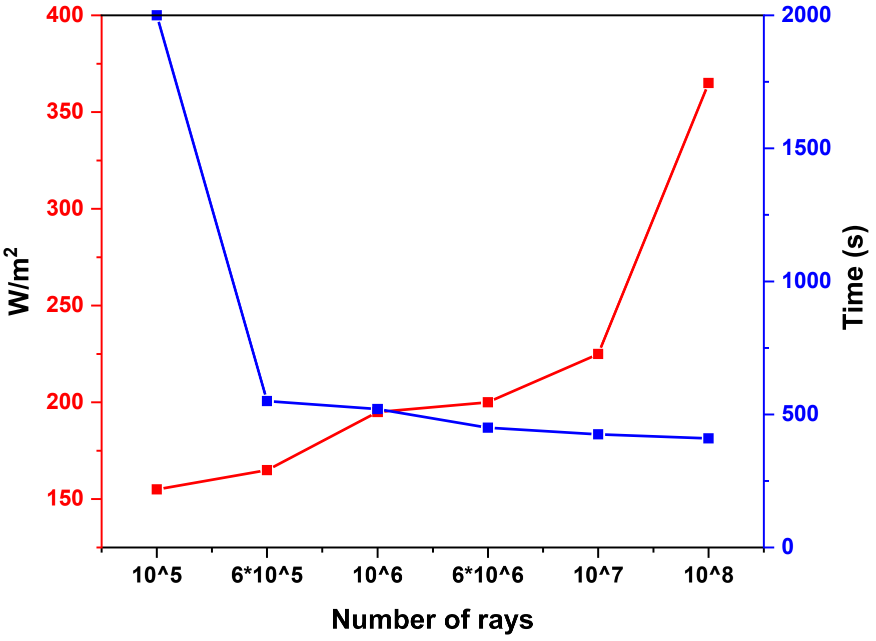

Figure S3. Definition of simulated rays' number.

To assess the impact of the number of traced rays on the simulation, both the simulation completion time and the standard deviation of the irradiance data set were analyzed. Figure S3 illustrates that an exponential increase in simulation time was observed as the number of processed rays increased. Conversely, the standard deviation demonstrated a tendency to stabilize around a value close to 200 W/m² as the number of processed rays increased. To strike a balance between simulation accuracy and time efficiency, a threshold of $6 \cdot 10^6$ rays was employed as the stopping criterion for the ray tracing simulations conducted in this research. This threshold was determined to provide a satisfactory level of accuracy while managing the computational time required.

S6. Chemical composition analysis for prepared membranes

From EDS surface analysis, $CoFe_2O_4$ elements were detected on the surfaces of the membranes showing that during phase inversion, the $CoFe_2O_4$ nanoparticles migrated to the membrane surface (**Figure S4b, c,** and **d**). From this observation, it was expected that membranes with higher loading would have higher photodegradation performance in comparison to loadings > 0.5 wt% $CoFe_2O_4$-PES membranes. This is because photodegradation mainly occurs on the surfaces of the membranes where the nanoparticles are fully exposed to light.

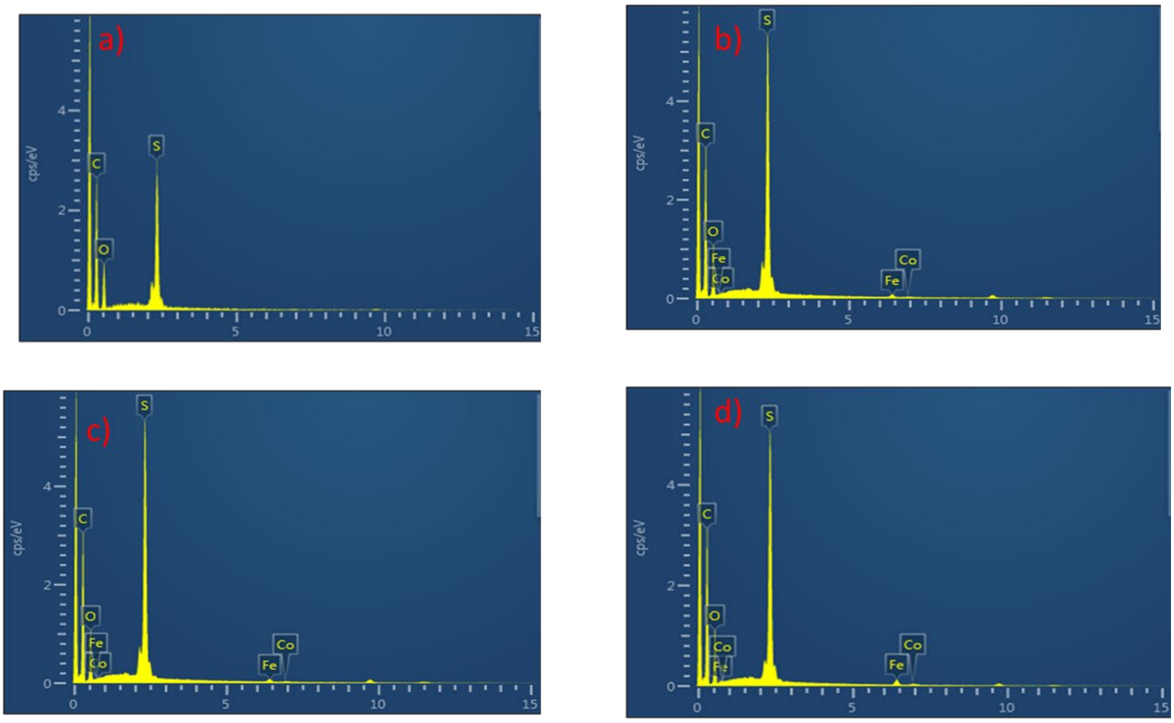

Figure S4. Chemical composition (EDS) for the fabricated membranes (M0(a), M1 (b), M2 (c), and M3 (d)): M0 with 0% $CoFe_2O_4$ nanoparticles; M1 with 0.2% $CoFe_2O_4$ nanoparticles; M2 with 0.5% $CoFe_2O_4$ nanoparticles and M4 with 1% $CoFe_2O_4$ nanoparticles.

S7. Contact angles

All membrane samples (M1-M3) with $CoFe_2O_4$ nanoparticles experienced a noticeable decrease in contact angle values as compared to pristine (M0) membranes (**Figure S5**). The decrease in contact angle indicated that $CoFe_2O_4$ nanoparticles improved the hydrophilicity of PES membranes and M3 that had $CoFe_2O_4$ loading of 1 wt.% was the most hydrophilic. Improvement in hydrophilicity was expected to enhance interactions between water and membranes during the photocatalytic degradation process and as a result, increase chances of collision between water molecules and holes to produce hydroxyl radical species subsequently improving the photodegradation process.

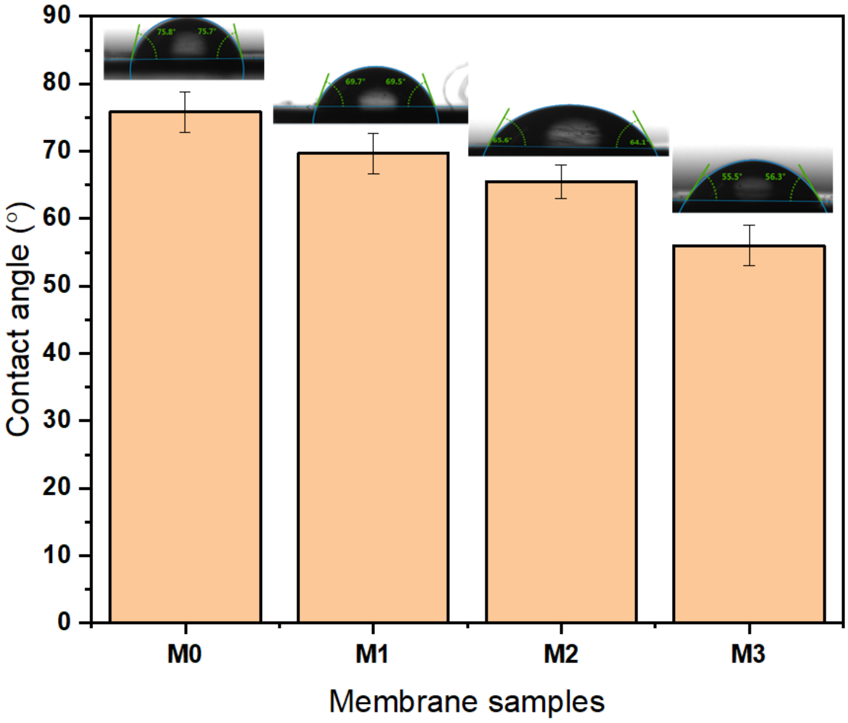

Figure S5. Water contact angle for pristine and $CoFe_2O_4$ modified membranes; M1 with 0.2% $CoFe_2O_4$ nanoparticles; M2 with 0.5% $CoFe_2O_4$ nanoparticles and M4 with 1% $CoFe_2O_4$ nanoparticles.

S8. Pure water flux.

Water flux can be used to evaluate the membrane permeation performance. From **Figure S6**, the water flux decreased with an increase in $CoFe_2O_4$ loading in the membranes and the flux was in the following order: M0 > M1 > M2 > M3. This was not expected as flux normally increases with the hydrophilic of membranes. In this study, pristine (M0) had the highest pure water permeability (L_p), which was 6 times higher than the membrane with the highest nanoparticle loading (i.e., M3 with 1wt % $CoFe_2O_4$ nanoparticles). Agglomeration of nanoparticles and pore blockage might be responsible for changes in pore size and bulk porosity of the membranes, which resulted in a decline in membrane pure water permeability (L_p) [3–8].

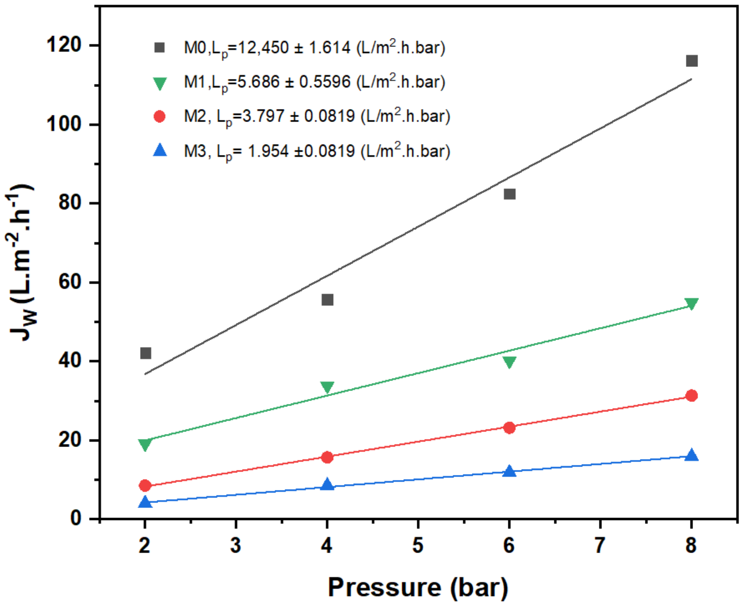

Figure S6. Water permeability of the prepared membranes;M0 pristine M1 with 0.2% $CoFe_2O_4$ nanoparticles; M2 with 0.5% $CoFe_2O_4$ nanoparticles and M4 with 1% $CoFe_2O_4$ nanoparticles.

S9. FTIR analysis of the prepared membranes

The surface functionalities of the membranes were analyzed to identify the functional groups at the surface of the membranes. The FTIR spectra for the membranes with peaks assigned to different functional units are shown in Figure S7. The presence of 2608 and 2350 cm^{-1} bands shows the existence of stretching and interaction between ethyl glycol and $CoFe_2O_{4-x}(OH)_x$. Moreover, the characteristic peaks registered at 2848 cm^{-1} is due to the symmetric stretching of C–H in alkyl chains from PES [9][10]. Absorption bands exhibited at 1486 and 1579 cm^{-1} were attributed to the vibration of -C=C bonds. Then, peaks 1142 and 1242 cm^{-1} were associated with the symmetric and asymmetric stretching vibrations of S– O from PES, respectively [11]. Aromatic stretching (C-O-C) was illustrated at a peak of 1230 cm^{-1}. All spectra had similar infrared absorption bands, suggesting that the structural bonds of PES were not altered by the addition of $CoFe_2O_4$ nanoparticles to the polymer matrix.

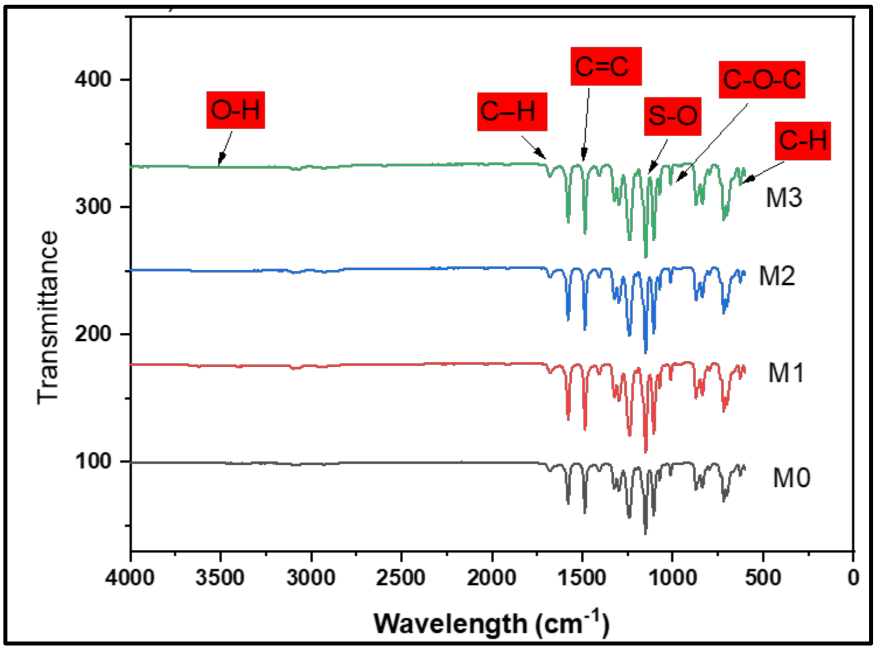

Figure S7. FTIR analysis of prepared $CoFe_2O_4$ -PES membranes.

Reference

1. S. Marais et al., Natural organic matter (nom) in south african waters volume i: nom fractionation, characterisation and formation of disinfection by-products A Report to the Water Research Commission. 2018. Available: www.wrc.org.za
2. V.M. ChakachakaO.T. MahlanguC.S. TshanganaB.B. MambaA.A. Muleja, Highly adhesive $CoFe_2O_4$ nanoengineered PES membranes for salts and Naproxen removal and antimicrobial activities, Journal of Membrane Science 676 (2023) 121612, https://doi.org/10.1016/j.memsci.2023.121612
3. S. Zinadini, A. A. Zinatizadeh, M. Rahimi, V. Vatanpour, H. Zangeneh, and M. Beygzadeh, "Novel high flux antifouling nano fi ltration membranes for dye removal containing carboxymethyl chitosan coated Fe 3 O 4 nanoparticles," *DES*, 349 (2014, https://doi.org/10.1016/j.desal.2014.07.007.
4. Y. Chen, F. Giralt, and Y. Cohen, "Hydraulic Resistance and Protein Fouling Resistance of a Zirconia Membrane with a Tethered PVP Layer, Water. 13(2021)951; https://doi.org/10.3390/w13070951
5. H. T. Dang, R. M. Narbaitz, T. Matsuura, and K. C. Khulbe, "A Comparison of Commercial and Experimental Ultrafiltration Membranes via Surface Property Analysis and Fouling Tests A Comparison of Commercial Water Quality Research J. 41 (2006)84–93. https://doi.org/10.2166/wqrj.2006.009and Experimental Ultrafiltration Membranes via Surface Property Analysis and Fouling Tests,"
6. O. T. Mahlangu, R. Nackaerts, B. B. Mamba, and A. R. D. Verliefde, "Development of hydrophilic GO-ZnO/PES membranes for treatment of pharmaceutical wastewater," Water Science and Technology, 76 (2017) 501–514, https://doi.org/10.2166/WST.2017.194.
7. R. Patala, O. T. Mahlangu, H. Nyoni, B. B. Mamba, and A. T. Kuvarega, "In Situ Generation of Fouling Resistant Ag / Pd Modified PES Membranes for Treatment of Pharmaceutical Wastewater," Membranes, 12(2022) 762; https://doi.org/10.3390/membranes12080762
8. O. T. Mahlangu, R. Nackaerts, J. M. Thwala, B. B. Mamba, and A. R. D. Verliefde, "Hydrophilic fouling-resistant GO-ZnO/PES membranes for wastewater reclamation," *J Memb Sci*, 524, (2017) 43–55, https://doi.org/10.1016/j.memsci.2016.11.018.
9. Sutisna, M. Rokhmat, E. Wibowo, Khairurrijal, M. Abdullah, Prototype of a flat-panel photoreactor using TiO2 nanoparticles coated on transparent granules for the degradation of Methylene Blue under solar illumination, Sustainable Environment Research. 27 (2017) 172–180. https://doi.org/10.1016/J.SERJ.2017.04.002.
10. B. Sahu, O. Fe, J. Siregar, N. Luh, W. Septiani, Effect of Template on Structural and Band Gap Behaviors of Magnetite Nanoparticles Effect of Template on Structural and Band Gap Behaviors of Magnetite Nanoparticles, Journal of Physics, 1093 (2018) 012020. https://doi :10.1088/1742-6596/1093/1/012020
11. C.S. Tshangana, A.A. Muleja, E.N. Nxumalo, S.D. Mhlanga, Poly (ether) sulfone electrospun nanofibrous membranes embedded with graphene oxide quantum dots with antimicrobial activity, Environmental Science and Pollution Research. 27 (2020) 26845–26855. https://doi.org/10.1007/S11356-020-09080.
